# High‐efficiency delivery of CRISPR‐Cas9 by engineered probiotics enables precise microbiome editing

**DOI:** 10.15252/msb.202110335

**Published:** 2021-10-19

**Authors:** Kevin Neil, Nancy Allard, Patricia Roy, Frédéric Grenier, Alfredo Menendez, Vincent Burrus, Sébastien Rodrigue

**Affiliations:** ^1^ Département de biologie Université de Sherbrooke Sherbrooke QC Canada; ^2^ Département de Microbiologie et d'Infectiologie Université de Sherbrooke Sherbrooke QC Canada

**Keywords:** antibiotic resistance, antimicrobials, bacterial conjugation, CRISPR‐Cas9, synthetic biology, Biotechnology & Synthetic Biology, Microbiology, Virology & Host Pathogen Interaction

## Abstract

Antibiotic resistance threatens our ability to treat infectious diseases, spurring interest in alternative antimicrobial technologies. The use of bacterial conjugation to deliver CRISPR‐*cas* systems programmed to precisely eliminate antibiotic‐resistant bacteria represents a promising approach but requires high *in situ* DNA transfer rates. We have optimized the transfer efficiency of conjugative plasmid TP114 using accelerated laboratory evolution. We hence generated a potent conjugative delivery vehicle for CRISPR‐*cas9* that can eliminate > 99.9% of targeted antibiotic‐resistant *Escherichia coli* in the mouse gut microbiota using a single dose. We then applied this system to a *Citrobacter rodentium* infection model, achieving full clearance within four consecutive days of treatment.

## Graphical abstract

A conjugative plasmid with high transfer rates is leveraged to deliver the CRISPR system into targeted bacteria. The resulting conjugative system can clear a *C. rodentium* infection in mice.

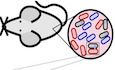

## Introduction

Rates of antibiotic‐resistant infections are rapidly increasing worldwide and threaten to become the second most important cause of mortality by 2050 (World Health Organization, 2014). *Enterobacteriaceae* are natural residents of the gut microbiota and are particularly infamous for their rapid accumulation of antibiotic resistance genes (Nordmann *et al*, 2011; Yamamoto & Pop‐vicas, 2014). This group comprises pathogens such as *Escherichia coli*, *Klebsiella pneumoniae*, *Shigella* sp., and *Salmonella* sp., which can cause deadly infections when not adequately treated (Falagas *et al*, 2014). Among these pathogens, *E. coli* is responsible for various diseases in farm animals and accounts for almost half of all human antibiotic‐resistant infections attributed to enterobacteria (Cassini *et al*, 2019).

The use of CRISPR‐Cas9 as an antimicrobial represents a promising strategy to precisely eliminate targeted bacterial populations. CRISPR‐Cas9 is a protein–RNA complex that can be programmed to cleave specific DNA sequences found only in target bacteria. The specificity of this system relies on the complementarity between a 20‐nucleotide sequence (spacer) present in a guide RNA (gRNA) and a target DNA sequence (protospacer) sitting next to a protospacer adjacent motif (PAM) (Koonin & Makarova, 2019). Upon detection of a valid protospacer in the genome of the target bacterium, the CRISPR‐Cas9 complex induces a double‐strand cut that fails to be repaired by non‐homologous end joining mechanisms as shown in *E. coli* (Mali *et al*, 2013; Cui & Bikard, 2016). CRISPR‐Cas9 cleavage of a chromosomal sequence leads to the death of the target bacterium (Gomaa *et al*, 2014; Cui & Bikard, 2016), whereas targeting a sequence found on a plasmid leads to its loss from the host cell (Dong *et al*, 2019; Neil *et al*, 2019). The key step to enable this technology for antimicrobial applications is to deliver the CRISPR‐*cas9* system to virtually all bacteria of the targeted population. Engineered bacteriophages have been proposed as delivery vehicles for CRISPR‐*cas9* systems, and proof‐of‐concept demonstrations have been performed in bacterial cultures as well as in mice models (Bikard *et al*, 2014b; Citorik *et al*, 2014; Yosef *et al*, 2015; Hsu *et al*, 2020). While bacteriophages offer interesting advantages such as high infectivity, they often display narrow host ranges (Yosef *et al*, 2015). Furthermore, phage receptors on the cell surface can quickly mutate (Shabbir *et al*, 2016) and environmental conditions (low pH, gastric fluid, proteases, etc.) encountered by viral particles can dramatically limit their activity in the gut (Nobrega *et al*, 2016).

**Figure 1:**
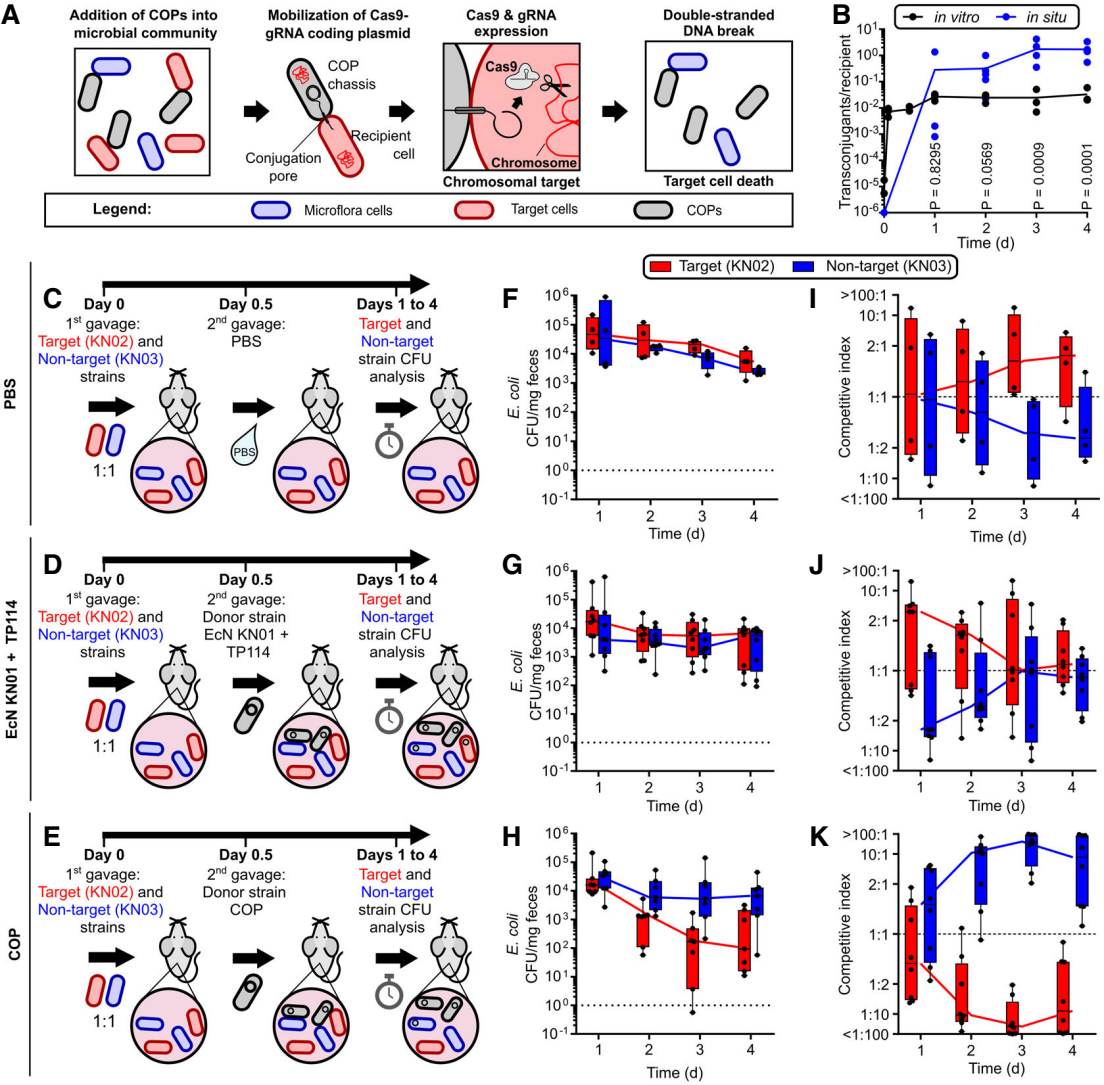
Conjugative probiotics (COPs) can transfer CRISPR‐*cas9* at high efficiency to specifically eliminate a target strain from the gut microbiota AMode of action of the COP system.BTransfer rates of TP114 on agar (*n* = 3, biological replicates), and in the murine gut (*in situ*) (*n* = 5 mice).C–EOverview of the mouse model experiments. Streptomycin‐treated mice were administered a 1:1 mixture of target and non‐target strains and subsequently treated with PBS (C), EcN KN01 + TP114 (D) or the COP (E).F–HAbsolute CFU abundance of the target and non‐target strain in mice feces after treatment with PBS (F), EcN KN01 + TP114 (G), or the COP (H). The dotted line indicates the highest detection limit based on feces weight (10^0^). A total of six mice were used for each experimental group (*n* = 6). All data points are shown.I–KCompetitive index of the target and non‐target strains calculated from the ratio of absolute CFU (see panels F–H) between both strains for the PBS (I), EcN KN01 + TP114 (J), or the COP (K) treated groups. The dotted line indicates the expected competitive index at equilibrium. Data information: (F‐K) Boxes show the 10–90 percentiles, and whiskers represent minimal and maximal values. The median is indicated by a line (*n* = 6 mice). Source data are available online for this figure.

Gut‐adapted bacteria such as probiotic strains could prove more resilient than bacteriophages within the intestinal microbiota and use bacterial conjugation to deliver CRISPR‐*cas9* to a broader range of target cells. Bacterial conjugation is a process involving a type IV secretion system (T4SS) to transfer DNA from a donor bacterium to a recipient cell in close physical contact, in some cases with high‐efficiencies (Arutyunov & Frost, 2013; Neil *et al*, 2020). Previous work provided evidences that conjugation can be repurposed to transfer CRISPR‐Cas modules between different bacterial species, achieving high efficiency in test tubes and offering important insights on the selection of spacers sequences (Hamilton *et al*, 2019; López‐Igual *et al*, 2019; Ruotsalainen *et al*, 2019). However, these same systems produced only modest effects in mice models, which would not be sufficient to reach clinical applications (Kamruzzaman *et al*, 2017; López‐Igual *et al*, 2019; Rodrigues *et al*, 2019; Neil *et al*, 2020). Thus, conjugative transfer rates have generally been considered to be limiting for this type of usage (Citorik *et al*, 2014). To overcome these limitations, our group has recently screened most conjugative plasmid families found in *Enterobacteriaceae* and identified plasmid TP114 as a highly proficient DNA transfer machinery in the gut microbiota (Neil *et al*, 2020). Here, we report the development of a genetically engineered conjugative probiotic (COP) strain that leverages TP114 for the mobilization of CRISPR‐*cas9* (Fig 1A). We demonstrate the feasibility of this approach by eliminating antibiotic‐resistant *E. coli* in the gastro‐intestinal (GI) tract and by treating a *Citrobacter rodentium* (CR) infection in a mouse model.

## Results

### COP strain design and construction

**Figure EV1:**
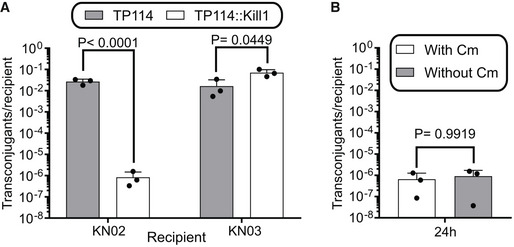
COPs can selectively eliminate a target strain from a mixed population *in vitro* Transfer frequency of TP114::Kill1 and TP114 from KN01∆*dapA* toward a 1:1 mix of target (KN02) and non‐target (KN03) recipient bacteria after 24 h.Selection of TP114::Kill1 transconjugants with or without chloramphenicol (Cm) allows the distinction between KN02 killing and Cm sensitization. Data information: Bars and error bars, respectively, show the mean and standard deviation of the mean of three biological replicates (*n* = 3). Statistical significance was determined using a one‐way ANOVA test on the log of the data. Source data are available online for this figure.

Several bacterial strains would constitute interesting bacterial chassis for the development of the COP system. Because of a long history of clinical use (Jacobi & Malfertheiner, 2011) and the availability of efficient genetic manipulation tools, we selected *E*. *coli* Nissle 1917 (EcN) as our initial testbed. Three EcN strains with different antibiotic resistance profiles were used (KN01, KN02, and KN03) to facilitate their discrimination in conjugation assays (Appendix Table S1). These three EcN derivatives all have Tn7‐mediated chromosomal insertion encoding streptomycin resistance as well as an additional unique resistance gene for each strain (KN01; spectinomycin, KN02; chloramphenicol, KN03; tetracycline). As previously reported, high *in situ* transfer rates were observed after the successive introduction in mice of recipient strain EcN KN02 followed by the donor strain EcN KN01 harboring the conjugative plasmid TP114, and by probing conjugation events in feces (Neil *et al*, 2020) (Fig 1B). The CRISPR‐*cas9* system was next armed with a gRNA targeting the chloramphenicol acetyl‐transferase (*cat*) gene inserted in the genome of EcN KN02. The resulting CRISPR‐*cas9* system formed a killing module (Kill1) that was integrated in TP114 by Double Recombinase Operated Insertion of DNA (DROID) (Neil *et al*, 2019) to generate TP114::Kill1 (Appendix Fig 1, Appendix Table S1). This specific *cis* system conformation allows for maximal efficiency as transconjugant cells can in turn disseminate the CRISPR module. The activity of TP114::Kill1 against EcN KN02 was tested *in vitro* on agar plates, using EcN KN03 as a control non‐target strain (Fig EV1A). While transfer frequencies of TP114::Kill1 in EcN KN03 were similar to the TP114 wild‐type plasmid, the rate of EcN KN02 + TP114::Kill1 transconjugant formation was > 10,000‐fold lower, suggesting that the CRISPR‐Cas9 system eliminated EcN KN02 containing the targeted *cat* gene upon DNA transfer. To discriminate the death of KN02 transconjugants from the loss of their chloramphenicol resistance, TP114::Kill1 was transferred again to the targeted KN02 strain and transconjugants were selected on plate with or without chloramphenicol (Fig EV1B). Transconjugants frequency remained similar in both conditions, suggesting that the transfer of TP114::Kill1 to the target strain resulted in the elimination of the bacterium rather than in the sensitization to chloramphenicol.

### The COP system as a microbiome editing tool

**Figure EV2:**
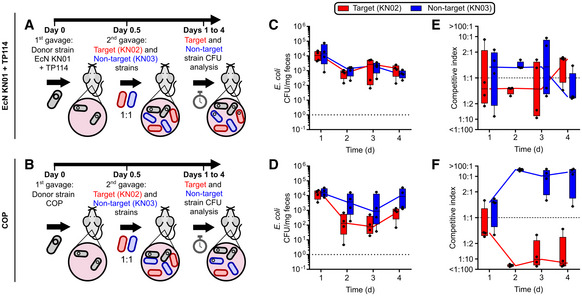
COPs can protect the microbiota from colonization of a specific strain A, BMouse model experiment. Mice either received a dose of an EcN KN01 + TP114 (A) or the COP (B) 12 h prior to the introduction of the target/non‐target 1:1 mix.C, DCFU abundance of the target and non‐target strains for the mice groups given EcN KN01 + TP114 (C) or the COP (D) followed in feces for up to 4 days post‐gavage (*n* = 4). The dotted line indicates the highest detection limit based on feces weight (10^0^).E, FCompetitive indexes calculated from the CFU ratio between both strains in mice treated prophylactically with either EcN KN01 + TP114 (e) or the COP (f) (*n* = 4). The dotted line highlights the expected competitive index at equilibrium. Data information: (C–F) For all box and whiskers plots, medians are shown as a bar, boxes represent the 10–90% percentile of the data, and whiskers extend to the minimal and maximal values. Source data are available online for this figure.

The activity of the COP strain EcN KN01 bearing TP114::Kill1 was next assessed in the mouse gut microbiota. Mice were first treated with 1 g/l of streptomycin in their drinking water to promote colonization of all introduced strains as previously reported (Schinner *et al*, 2015; Neil *et al*, 2020). Mice then received a ˜1 × 10^8^ colony‐forming unit (CFU) 1:1 mixture of EcN KN02 (target) and EcN KN03 (non‐target) by gavage to test both the efficiency and specificity of killing. Twelve hours later, a second gavage was performed using phosphate buffered saline (PBS), ˜1 × 10^8^ CFU of EcN KN01 + TP114, or ˜1 × 10^8^ CFU of the COP strain (Fig 1C–E). The amount of EcN KN02 and KN03 was then quantified in feces over a period of 4 days without any additional administration of the treatments (Fig 1F–H). No significant difference was observed in the ratio of the EcN recipient strains KN02 and KN03 for the PBS and TP114 controls (Fig 1I and J). However, the number of EcN KN02 target bacteria was decreased by almost two orders of magnitude in the presence of the COP (median = 98.6% decrease 36 h after the administration of the COP) (Fig 1K). Similar results were obtained when the COP strain was introduced as a prophylactic treatment 12 h prior to the gavage of the target and non‐target strains, leading to a specific ˜30‐fold depletion of the targeted KN02 strain (median = 96.4% decrease 36 h after the administration of the COP) (Fig EV2). As previously reported (Reuter *et al*, 2021), escaper mutants, which are target bacteria that received the CRISPR module but were not eliminated, can be detected at low frequencies during *in vitro* (Appendix Fig S2A) and *in situ* (Appendix Fig S2B) experiments. Sequencing these escapers revealed deleterious mutations of various types in either the gRNA or *cas9* gene (Appendix Fig S1C and D).

### Metagenomic evaluation of the COP system accuracy

To evaluate the broader impact of the COP strategy on the microbiota, 16S ribotyping was performed using feces from the previous experiments. In this streptomycin‐treated model, Shannon alpha diversity was highly similar between treatments (average Kruskal–Wallis *P* = 0.8130), indicating that the COP had no significant effect on the microbiota diversity of each sample (Appendix Fig S3A). Moreover, weighted UniFrac beta diversity for both the COP or the EcN KN01 bearing TP114 control strain were not significantly different, suggesting that the treatment with the COP did not induce major shifts in microbiota composition as compared to the control groups (Appendix Fig S3B–H). No bias in the variation of the relative abundance of 10 highly conserved bacterial groups could be detected in the COP‐treated samples relative to controls, indicating that no significant off‐targeting activity affected their abundances (Appendix Fig S3I). Taken together, these results show that a single dose of the COP can efficiently decrease the abundance of a target strain *in situ*, leaving the rest of the microbiota essentially untouched.

### DNA delivery system optimization by accelerated laboratory evolution

**Figure 2:**
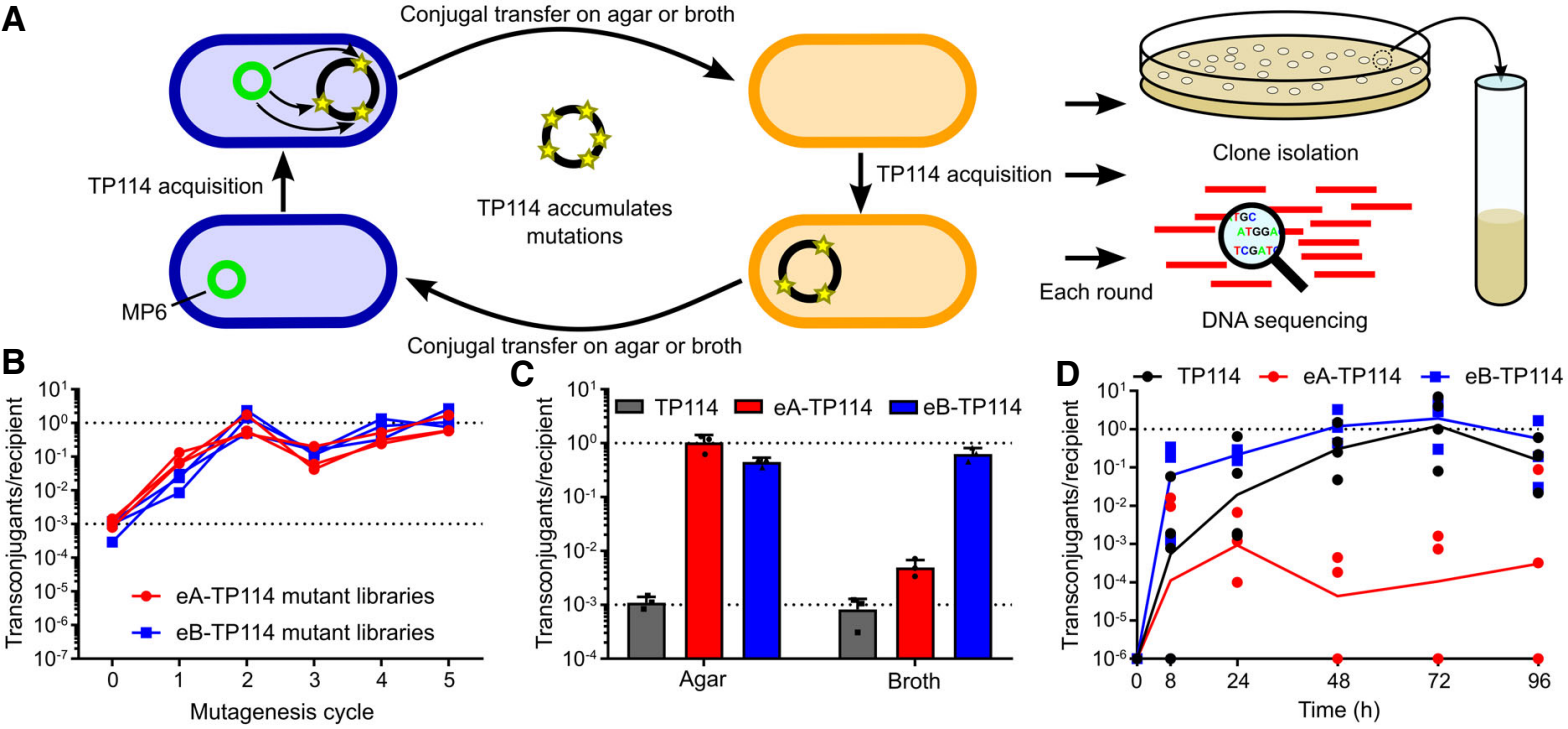
Improvement of TP114 transfer rates by accelerated laboratory evolution Schematic representation of the experiment.TP114 mutant libraries transfer rates after 2 h at 37°C measured after each round of random mutagenesis on agar (eA) or in broth (eB) (*n* = 3). The lower dotted line indicates wild‐type TP114 average transfer rate (˜10^−3^), whereas the higher dotted line highlights the maximal transfer rate (10^0^).Comparison of conjugation efficiencies on agar or in broth for wild‐type TP114 as well as the evolved eA‐TP114 and eB‐TP114 mutants obtained after five rounds of mutagenesis. Histograms present the average transfer rates and error bars represent standard deviation (*n* = 3, biological replicates). All data points are shown. The lower dotted line indicates wild‐type TP114 average transfer rate (˜10^−3^), whereas the higher dotted line highlights the maximal transfer rate (10^0^).Comparison of TP114, eA‐TP114, and eB‐TP114 transfer rates *in situ* quantified in feces. Four streptomycin‐treated mice were used for each experimental group (*n* = 4). The dotted line highlights the maximal transfer rate (10^0^). Source data are available online for this figure.

**Figure EV3:**
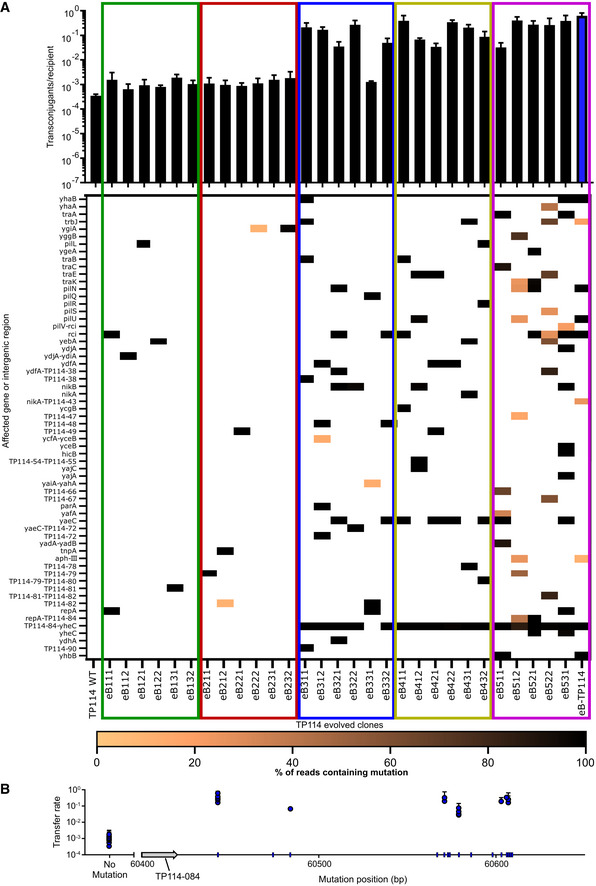
Correlation between mutation location and transfer rates of TP114 evolved clones Transfer rates of wild‐type TP114 in broth compared to 30 clones of TP114 evolved for conjugation in broth (eB) and isolated at different mutagenesis cycles (*n* = 3, biological triplicate). The transfer rate of each clone is shown above the heatmap representing its mutation profile, with the color scale representing the proportion of reads confirming the presence of a variant. Bar and error bars represent the average and standard deviation of the data. The blue bar highlights the conjugation rates of the eB‐TP114 selected for the eB‐COP system (eB527).Mutation positions and associated transfer rates for the intergenic region located between TP114‐084 and TP114‐085. Transfer rates of clones for which no mutation was detected in this region were grouped under “no mutation”. The blue tick marks on the x‐axis indicate the position of mutations found in the intergenic region between TP114‐084 and TP114‐085 in all clones presented in Appendix Fig S6. Clones are named as described in Appendix Fig S6. Error bars represent the standard deviation of the data points. Source data are available online for this figure.

We next reasoned that accelerated laboratory evolution (ALE) could be applied to the generation of more efficient variants of TP114. Plasmid MP6 was used to randomly introduce mutations in TP114 (Badran & Liu, 2015) hosted in the diaminopimelic acid (DAP) auxotroph EcN KN01Δ*dapA*. This step generated a library of donor strains containing mutated copies of TP114 that were used in conjugation experiments either on agar or in broth. TP114 variants first competed for transfer to the recipient EcN KN03 strain, and the EcN KN01Δ*dapA* donor was counter‐selected in the absence of DAP supplementation. TP114 plasmid mutants isolated in EcN KN03 transconjugants were then re‐introduced into EcN KN01Δ*dapA* + MP6 for five consecutive rounds of mutagenesis (Fig 2A). The transfer rates of the mutant libraries were measured at each round (Fig 2B) and improved by three orders of magnitude to reach nearly 100% at the end of the experiment. Mutations acquired by TP114 during ALE were identified for bulk mutant libraries as well as for six or seven isolated clones per round of mutagenesis evolved for transfer on agar or in broth (eA or eB, respectively) (Appendix Fig S4 and Dataset EV1). Individual eA and eB strains were named using a three‐digit suffix, respectively corresponding to the mutagenesis round, the replicate, and the clone number (Appendix Figs S5 and S6). While ALE induced a variety of mutations, a gradual enrichment for mutations in the intergenic region between TP114‐084 and TP114‐085 was observed for eA‐ and eB‐TP114 libraries, which was highly correlated with increased transfer rates (*P*‐value = 1.47 × 10^−42^) (Fig EV3). eB‐TP114 mutants were additionally enriched with mutations in *yaeC* (TP114‐70), a predicted transcription regulator encoded by TP114. Conversely, eA‐TP114 mutants displayed an increased number of deleterious mutations in the *pil* genes compared to eB‐TP114 (including 8 high and 12 moderate impact mutations, see Dataset EV1), which are required for the assembly of the Type IV Pilus (T4P), supporting the importance of the T4P for conjugation in broth (Ishiwa & Komano, 2000; Neil *et al*, 2020).

### Highly efficient DNA delivery by eB‐TP114

Two representative clones, eA‐TP114 (eA517) and eB‐TP114 (eB527), were next selected to systematically quantify transfer frequencies on agar, in broth, and in the mouse GI tract. Both eA‐TP114 and eB‐TP114 transferred at > 400‐fold higher rates on agar relative to wild‐type TP114 (Fig 2C). On the other hand, eA‐TP114 showed only a modest increase in transfer rates in broth (˜6‐fold increase), while eB‐TP114 improved by almost three orders of magnitude (˜768‐fold) compared to TP114. Conjugative transfer in the mouse gut microbiota was next assessed by administering the recipient EcN KN02 strain by gavage, followed 2 h later by the EcN KN01 donor strain carrying TP114, eA‐TP114 or eB‐TP114. Not surprisingly, eA‐TP114 showed relatively low conjugation rates due to the presence of a mutation in the T4P, confirming its involvement in mating pair stabilization *in situ* (Neil *et al*, 2020) (Fig 2D). In contrast, the eB‐TP114 clone reached higher transfer efficiencies, with faster kinetics than TP114. For instance, an average of ˜17% of the target cells had received eB‐TP114 only 8 h after gavage of the donor strain while this took between 24 and 48 h for TP114.

### Efficient elimination of an antibiotic‐resistant *E. coli* population in the mouse gut

**Figure 3:**
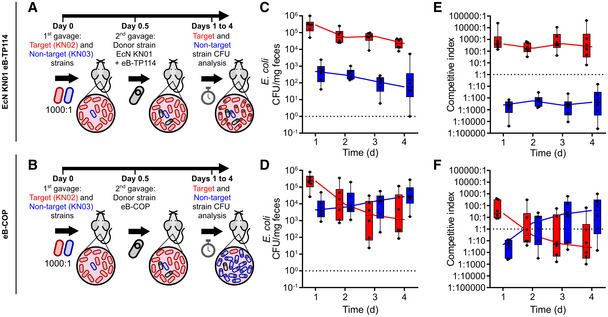
The eB‐COP system can reshape bacterial populations in the gut A, BSchematic representation of the experiment. Streptomycin‐treated mice were first administered with a 1,000:1 mix of target/non‐target strains and were treated 12 h later with either EcN KN01 + eB‐TP114 (A), or the eB‐COP (B).C, DAbsolute CFU abundance of the target and non‐target strain monitored in feces of mice treated with EcN KN01 + eB‐TP114 (C) or the eB‐COP (D). The dotted line indicates the highest detection limit based on feces weight (10^0^). A total of six mice were used for each experimental group (*n* = 6). All data points are shown.E, FCompetitive indexes of the target and non‐target strains followed daily with EcN KN01 + TP114 devoid of a CRISPR‐Cas9 system (E) and in mice treated with the eB‐COP system (F). The dotted line indicates the expected competitive index at equilibrium. Data information: (C–F) Boxes show the 10–90 percentiles, and whiskers represent minimal and maximal values. The median is indicated by a line (*n* = 6 mice). Source data are available online for this figure.

**Figure EV4:**
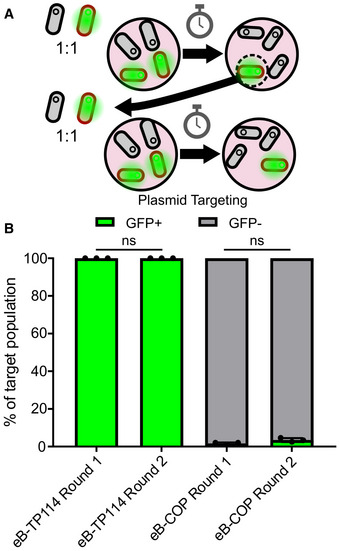
The remaining fraction of target bacteria after eB‐COP remains susceptible to the system Overview of the re‐exposition experiment. A recipient strain carrying a GFP plasmid encoding the targeted *cat* gene was mixed with the eB‐COP strain. After a first round of plasmid elimination, three residual GFP‐positive colonies were selected for a re‐challenge with a second round of eB‐COP.Plasmid elimination measured by counting the proportion of GFP positive (GFP^+^) and GFP negative (GFP^−^) colonies after one or two successive rounds of COP treatment. As a control, the same strain was exposed to KN01 carrying eB‐TP114 without a CRISPR module. All experiments were performed in biological triplicates (*n* = 3), and error bars represent the standard deviation of the data points. Source data are available online for this figure.

Based on the higher transfer efficiency of eB‐TP114 *in situ*, we sought to exploit this variant as an improved version of the conjugative machinery for the COP system. The CRISPR‐*cas9* killing module was introduced in eB‐TP114, producing the eB‐COP strain (Appendix Table S1). To challenge the eB‐COP system, ˜1 × 10^8^ CFU of the target (KN02) and ˜1 × 10^5^ CFU of the non‐target (KN03) EcN bacteria were orally administered to streptomycin‐treated mice in a 1,000:1 (target:non‐target) ratio. Twelve hours later, ˜1 × 10^8^ CFU of the EcN KN01 carrying eB‐TP114 control strain (devoid of the CRISPR‐*cas9* system) or ˜1 × 10^8^ CFU of the eB‐COP strain was introduced by gavage (Fig 3A and B). The colonization levels of the target (KN02) and non‐target (KN03) strains were next followed in feces 24, 48, 72, and 96 h after their introduction in mice (Fig 3C and D). While the populations of EcN KN02 and KN03 showed slight decreases over time, their competition indexes remained constant in the EcN KN01 eB‐TP114‐treated groups (Fig 3C and E). In contrast, a complete shift in bacterial populations was observed in eB‐COP‐treated mice (Fig 3D and F). As little as 12 h after the introduction of the donor strain, eB‐COP was able to reduce the population of EcN KN02 relative to KN03 by ˜95% compared to the control. On day 2, the chloramphenicol‐resistant strain KN02 no longer accounted for the majority of the population in eB‐COP‐treated mice, with an average elimination of 99.5%. Four days after the single administration of eB‐COP, the treatment achieved a decrease of > 4,000‐fold of the mean EcN KN02 CFU relative to the corresponding eB‐TP114 control group, reaching a clearance of > 99.9% of the targeted bacteria. By specifically targeting the *cat* gene of an *E*. *coli* strain introduced in the gut, the eB‐TP114::Kill1 system completely shifted the composition of the *E. coli* population from a largely chloramphenicol‐resistant state to an almost totally sensitive one. No resistance to the CRISPR module was detected when probing for bacteria harboring the combined resistance markers found on TP114 and in EcN KN02, indicating that all target cells that received eB‐TP114::Kill1 were eliminated (resistance frequency is below the detection limit of ˜10^−6^). The remaining < 0.1% of target bacteria survived but did not acquire TP114::Kill1 based on their antibiotic resistance profile, raising the possibility that they could have been recalcitrant to conjugative transfer. To investigate this possibility, we performed an *in vitro* experiment by mixing the eB‐COP with EcN KN03 harboring a GFP reporter plasmid pT containing the targeted *cat* gene. After 24 h, ≥ 98% of EcN KN03 had lost the targeted plasmid but three GFP‐positive clones were recovered and re‐exposed to the eB‐COP (Fig EV4). A similar level of plasmid elimination was observed during this second treatment, suggesting that spared EcN KN03 did not possess inheritable resistance to bacterial conjugation and were likely not reached by the eB‐COP strain. Residual target bacteria may therefore become actionable with further improvements to the system or to the eB‐COP administration regimen.

### eB‐COP recognition of a variety of *E. coli* strains

**Figure 4:**
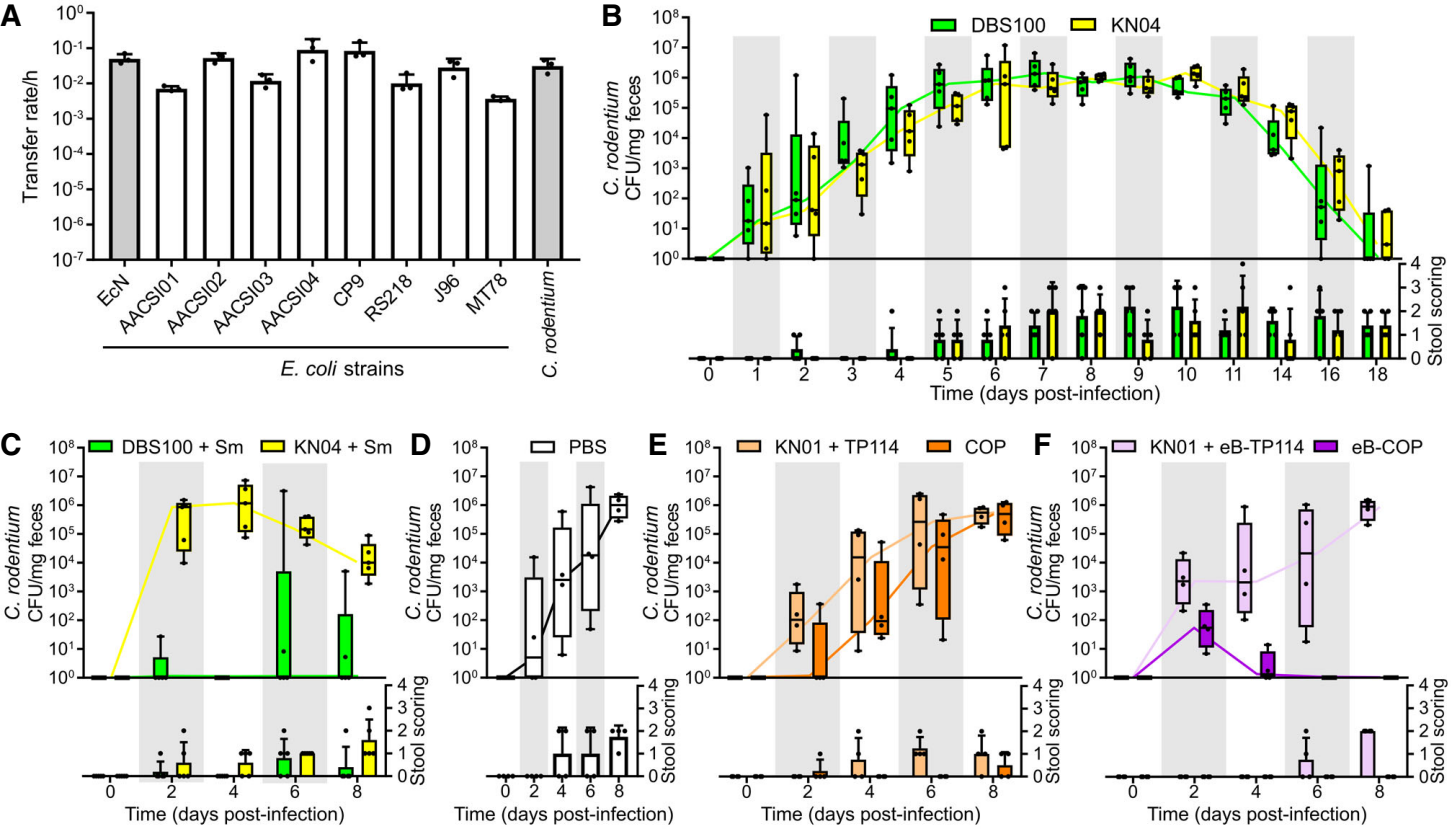
eB‐COP can transfer DNA to clinically relevant *E. coli* strains and eliminate *C*. *rodentium* from infected mice ATransfer rates of eB‐TP114 measured after 1 h of conjugation in broth. *E. coli* Nissle 1917 (EcN) and *C. rodentium* (CR) DBS100 are highlighted in gray. *E. coli* strains used as recipients are described in Appendix Table S1. Histograms show the average of the data and error bars represent the standard deviation calculated from three biological replicates (*n* = 3).BColonization levels of CR DBS100, and its streptomycin/chloramphenicol resistant variant KN04 as quantified in feces. Data include five mice per group, and individual datapoints are shown as dots on graphs (*n* = 5 mice).CQuantification of streptomycin‐sensitive CR DBS100 parental strain or its resistant variant KN04 in the feces of mice treated with 1 g/l of streptomycin in their drinking water from day 1 to 8 post‐infection (*n* = 5 mice).DColonization levels of CR KN04 in infected mice treated daily 24 h post‐infection with 100 µl of PBS from day 1 to 7 (*n* = 4 mice).ECR KN04 quantified in feces of mice that received either KN01 + TP114 or the COP. ˜1 × 10^8^ CFU of bacteria was administered by gavage daily for seven consecutive days starting 24 h post‐infection (*n* = 4 mice).FCR KN04 quantified in feces of mice that received either the evolved versions KN01 + eB‐TP114 or the eB‐COP. ˜1 × 10^8^ CFU of bacteria was administered by gavage daily for seven consecutive days starting 24 h post‐infection (*n* = 4 mice).B–FLower panel shows mouse stool scoring at each timepoint. Stool scoring was performed as part of a blind study design and relied on pre‐defined criteria. A stool score of 0 is attributed to normal feces, 1: presence of mucus, 2: soft feces, 3: liquid feces, 4: presence of blood in the feces. Data information: Boxes show the 10–90 percentiles, and whiskers represent minimal and maximal values. The median is indicated by a line. Source data are available online for this figure.

The ability of eB‐TP114 to recognize and transfer to clinically relevant *E. coli* strains was next investigated. A group of nine *E. coli* strains isolated from humans and animals was used as recipients for a 1‐h conjugation assay in broth (Fig 4A, Appendix Table S1). Plasmid eB‐TP114 transferred to all strains at relatively high rates per hour. Interestingly, eB‐TP114 also transferred to *C*. *rodentium* (CR) strain DBS100 at high rates, raising the possibility that the eB‐COP might be able to eliminate other bacterial species related to *E. coli*. Assuming that the average conjugation efficiency observed with these strains is constant (transfer rate of 2.4 ± 4.1 × 10^−2^/h), the majority of bacteria in a targeted population would be eliminated within approximately 2 days of eB‐COP treatment.

### Impact of eB‐COP on CR mouse infection model

The CR infection model in C57BL/6 mice is commonly used to simulate and study the virulence of enteropathogenic and enterohemorrhagic *E. coli* strains (EPEC, EHEC) (Crepin *et al*, 2016). A streptomycin resistance marker and the CRISPR‐targeted chloramphenicol resistance genes were introduced in the chromosome of CR DBS100 at a neutral site near the *glmS* gene to generate CR KN04, hence facilitating strain discrimination and causing susceptibility to eB‐COP. The virulence of the CR KN04 and DBS100 strains was next compared in a C57BL/6 mouse infection model. Interestingly, since robust colonization of the mouse gut microbiota was achieved using CR, no streptomycin treatment was necessary for these experiments. Each mouse received ˜1 × 10^8^ CFU of either strain by gavage, and the presence of CR was monitored in feces every day (Fig 4B). Both strains induced typical symptoms (diarrhea and mild weight loss) with similar colonization levels and infection dynamics as described in the literature (Crepin *et al*, 2016), suggesting that the genomic modification of CR KN04 had no impact on virulence. As a reference for antibiotic treatment efficiency, mice were infected with sensitive (DBS100) or resistant (KN04) CR strains, and streptomycin (1 g/l) was added in their drinking water on the next day for the rest of this experiment only (Fig 4C). CR was monitored in feces, revealing that streptomycin was able to virtually clear the sensitive CR DBS100 parental strain in about 4 days although resistance arose 2 days later in half of the mice. As expected, the streptomycin treatment failed to eradicate the resistant CR KN04 strain from infected mice, which showed bacterial loads in their feces similar to the untreated control groups (Fig 4B).

The ability of the COP system to fight the infection by the antibiotic‐resistant CR KN04 strain was next assessed. Mice were first infected with ˜1 × 10^8^ CFU of CR KN04 by oral gavage. Starting 1 day post‐infection, mice groups received either PBS, ˜1 × 10^8^ CFU of EcN KN01 + TP114, or ˜1 × 10^8^ CFU of the COP for the next 7 days (Fig 4D and E). This administration regime was adopted to ensure long‐lasting effects of the treatment in this context as EcN fails to stably colonize mice without streptomycin treatment (Neil *et al*, 2020). Colonization levels of CR as well as typical symptoms associated with the infection were evaluated on day 2, 4, 6, and 8 post‐infection. The EcN KN01 + TP114 strain had no significant effect on the observed symptoms or the CR bacterial load when compared to the PBS‐treated group. On the other hand, the COP reduced the symptoms experienced by the mice but only had a transient impact on CR bacterial burden. Compared to the EcN KN01 + TP114 control, the COP reduced the CR abundance in feces by 96% on day 2 post‐infection, but this effect gradually decreased and almost vanished after 8 days.

We surmised that the eB‐COP system, with its faster transfer kinetics, might show improved elimination of CR. Mice were infected with CR and treated for seven consecutive days with either EcN KN01 + eB‐TP114 or the eB‐COP (Fig 4F). While the EcN KN01 + eB‐TP114 control strain devoid of the CRISPR system did not produce any effect on CR KN04 colonization levels, the evolved eB‐COP system was able to clear CR from mice within 4 days of treatment. These high levels of strain elimination produced by the eB‐COP also prevented any symptom to arise in this group of mice. Taken together, these results showed that repeated treatments of eB‐COP produced bactericidal activity against CR that was similar to streptomycin, although without any resistance observed.

## Discussion

Different strategies have been proposed to develop new technologies that could replace antibiotics. The use of a programmable nuclease such as CRISPR‐Cas is particularly interesting to eliminate target bacteria based on their genomic sequences. However, high‐efficiency delivery of CRISPR‐Cas into the target bacteria is an essential step for this strategy to work. While bacteriophages have been proposed for this task, bacterial conjugation has generally been dismissed since transfer rates were considered too low to offer a viable solution (Bikard *et al*, 2014a; López‐Igual *et al*, 2019). Our work provides a clear demonstration that bacterial conjugation can be leveraged for efficient CRISPR‐Cas delivery. In our experiments, the eB‐COP prototype even proved as effective as streptomycin to clear CR from infected mice (Fig 4C and F).

In contrast to most antibiotics (Langdon *et al*, 2016), the use of CRISPR‐Cas9 as a killing agent promises a unique surgical precision to the treatment, ensuring that the targeted bacteria are eliminated with minimal impact on the rest of the microbiota. Our results support this hypothesis since no significant changes were detected in the major bacterial orders surveyed by metagenomics (Appendix Fig S3). However, the impact of the COP strategy on the microbiota might differ depending on the selected gRNA sequences. Several groups have developed spacer design algorithms to maximize on‐target activity and help limit off‐targeting (Guo *et al*, 2018; Calvo‐Villamañán *et al*, 2020; Reuter *et al*, 2021). By adapting these strategies to consider the entire composition of the microbiome, one could in principle design gRNA to specifically eliminate specific bacterial strains or species from the microbiota.

The present study focused primarily on the optimization of the DNA transfer machinery to maximize treatment efficiency. Thus, only a single gRNA targeting the *cat* gene, with an easily assessable chloramphenicol resistance phenotype, was used to isolate conjugation as the main variable affecting treatment efficiency. While targeting antibiotic resistance genes could be an attractive strategy for certain applications, this approach might not be the best option to ensure antibacterial activity as antibiotic resistance genes are often found on extra‐chromosomal DNA molecules (Norman *et al*, 2009) that could simply be lost without killing the host bacterium. Targeting conserved essential genes located in the chromosome (e.g., ribosomal proteins) would maximize the chances of triggering cell death (Cui & Bikard, 2016; Hamilton *et al*, 2019).

With the ability to tune the transfer spectrum of TP114 or other conjugative plasmids, one could envision using the COP system as a customizable microbiome editing technology. For example, bacterial species associated with specific diseases such as adherent invasive *E*. *coli* (AIEC) in Crohn’s disease‐affected patients (Palmela *et al*, 2018) or the increased abundance of *Firmicutes* in obese individuals (Turnbaugh *et al*, 2006) could be knocked down using the COP approach. Another possibility would be to program the COP to remove antibiotic resistance genes from all residents of the gut microbiome in an effort to revitalize conventional antibiotics (Van Schaik, 2015). The host range of conjugative plasmids was also reported to be larger than bacteriophages (Jain & Srivastava, 2013), which represents a considerable advantage for versatile DNA mobilization. However, additional work will be needed to better define or engineer the host range of TP114 and determine the breadth of applications for the COP approach.

ALE has proven to be a valuable tool to artificially enhance conjugation rates. Mutations found in the intergenic region between genes TP114‐084 and TP114‐085 appear to be key to the improvement of TP114 transfer rates (Appendix Figs S3–S5 and Fig EV3). Both TP114‐084 and TP114‐085 are located on the positive strand of DNA and encode proteins of unknown function. The implication of these genes for TP114 conjugation will require more investigation. The presence of mutations in the 084‐085 intergenic region might affect a regulatory element, leading to the deregulation of the downstream genes (from TP114‐085 to TP114‐005). Indeed, most of the mutations found in this region were either located within putative Dam methylation sites (GATC) or in a putative FNR binding site, a global regulator of transcription implicated in anaerobic gene regulation (Tolla & Savageau, 2010) (Dataset EV1). As TP114 regulation is affected by the presence of oxygen (Neil *et al*, 2020), the implication of FNR and Dam in the regulation of TP114 represents interesting avenues for future work on transcriptional regulation in TP114. It is likely that at least one of the genes located downstream TP114‐084 is involved in global plasmid regulation. We have also observed mutations in putative transcription regulator *yaeC* (TP114‐069) that could contribute to higher transfer rates.

We have observed a striking difference in the treatment of the CR infection between the COP and eB‐COP treatments (Fig 4E and F). While the COP initially lowered the abundance of CR in feces, this effect gradually disappeared. In contrast, the eB‐COP completely cleared the infection after 4 days. This was surprising given the relatively small difference in the killing efficiencies between the COP and eB‐COP (91.9% versus 99.98% after 4 days). We estimate that this phenomenon is likely due to the faster CR elimination kinetics mediated by the eB‐COP, which results in a rapid targeting of CR due to a mutation that likely derepressed TP114 conjugation (Fig 2D). The COP would require more time to reach high transfer efficiencies in response to low oxygen conditions or other environmental signals experienced in the gut. Given the difficulty of EcN to persist in the mouse GI tract in absence of streptomycin treatment (Neil *et al*, 2020), especially when symptoms such as diarrhea developed, the COP may not have sufficient time to achieve high conjugative transfer rates, hence compromising the treatment. The eB‐COP with its increased transfer rates would have a more decisive impact on the infection.

Our work provides a clear proof‐of‐concept that conjugation can be used to efficiently deliver genetic payloads such as the CRISPR system to bacterial residents of the intestinal microbiota. However, biocontainment measures will have to be implemented prior to potential COP usage in clinical applications. For instance, these measures will have to ensure that both the COP bacterium and the transferred DNA molecules will not persist in the microbiota or in the environment. Common strategies such as the generation of auxotrophic donor strains (Steidler *et al*, 2003; Allard *et al*, 2015) and *in trans* mobilization (Bikard *et al*, 2014b; Hamilton *et al*, 2019) of CRISPR‐encoding plasmids could be exploited. These types of modifications to the COP system will require to precisely localize critical DNA loci such as the origin of transfer and the origin of replication of TP114. Future studies will also have to evaluate the impact of potential barriers to DNA transfer such as plasmid exclusion and restriction‐modification systems on COP treatment efficiency, to systematically investigate the DNA transfer spectrum of conjugative plasmids and to study the COP treatment dynamics for this technology to mature toward potential applications in animal or human health. The COP could thus offer a new precision medicine strategy to prevent or treat a variety of microbiome‐related diseases and antibiotic‐resistant infections.

## Materials and Methods

### Strains, plasmids, and growth conditions

Strains and plasmids are detailed in Appendix Table S1 and are available upon request. Cells were routinely grown at 37°C for up to 18 h in Luria broth Miller (LB) or on LB agar medium supplemented with antibiotics when needed. Antibiotics were used at the following working concentrations: ampicillin 100 µg/ml, chloramphenicol 34 µg/ml, kanamycin 50 µg/ml, nalidixic acid 4 µg/ml, spectinomycin 100 µg/ml, streptomycin 50 µg/ml, tetracycline 15 µg/ml. DAP auxotrophy was complemented by adding DAP at a final concentration of 57 µg/ml in the medium.

### DNA manipulations

The EZ10‐Spin Column Plasmid Miniprep kit (Bio Basic) was used to extract small DNA vectors, whereas conjugative plasmids and genomic DNA were extracted using the Quick‐gDNA miniprep (Zymo Research) according to the manufacturer’s instructions. PCR amplifications were performed using Veraseq DNA polymerase (Enzymatics). Preparation of DNAseq libraries was performed using the NEBNext Ultra II FS DNA Library Prep Kit for Illumina (NEB). Purification of DNA was performed between each step of DNAseq and plasmid assembly using Agencourt AMPure DNA XP DNA binding beads (Beckman Coulter) following the manufacturer’s guidelines. After purification, DNA concentration and purity were routinely assessed using a NanoDrop spectrophotometer (Thermo Fisher Scientific).

### DNA transformation into *E. coli* by electroporation

Routine plasmid transformations were performed by electroporation. Briefly, to prepare electrocompetent *E. coli* strains, an overnight culture of the desired host strain was subcultured 1/50 in 20 ml of LB broth and allowed to grow until an optical density of 0.6 at 600 nanometers (OD_600nm_) was reached. Cells were then washed three times in sterile 10% glycerol solution and resuspended in 200 µl of 10% glycerol. The DNA to transform was then added to 40 µl of electrocompetent cells, which was then transferred in a 1‐mm electroporation cuvette. A pulse of 1.8 kV, 25 µF, and 200Ω for 50 ms was applied to the cells for electroporation. Cells were then resuspended in 1 ml of non‐selective LB medium and incubated 1 h at 37 or 30°C (for thermosensitive plasmids) for recovery before plating on selective media.

### *In vitro* conjugation assays

*In vitro* conjugation assays routinely used EcN KN01Δ*dapA* as the donor strain and EcN KN02 as the recipient strain unless specified otherwise. Bacterial cultures were grown for no more than 18 h prior to conjugation experiments. For each of the donor and recipient strain cultures, an adjusted volume of 500 µl at an OD_600nm_ of 1.0 was transferred to separate sterile 1.5‐ml microtube. Cells were centrifuged at 20,000× *g* for 1 min and washed once in 200‐µl sterile LB. The cells were then either resuspended in 2.5 µl (for conjugation on agar) or 500 µl (for conjugation in broth). Donor and recipient strains were next mixed at a 1:1 ratio. For conjugation on agar, the mixture was then deposited on an LB agar plate with DAP when appropriate. For conjugation in broth, the mixture was supplemented with DAP when appropriate and the microtube was put on a tube tumbler rotating mixer (VWR CAT# SBS550‐2) at 20 rpm. Conjugation mixtures were incubated at 37°C for 2 h, unless specified otherwise, before being resuspended in sterile PBS and diluted 1/10 serially. A volume of 5 µl of each dilution was then spotted in duplicate on LB selective plates to select donors, recipients, and transconjugants. All conjugation frequencies were reported as a factor of the recipient CFUs. All conjugation experiments were repeated in at least three independent biological replicates.

For successive plasmid targeting experiment, EcN KN03 was transformed with pT, a plasmid encoding the targeted *cat* gene, and an sfGFP reporter system. The EcN KN03 + pT strain was next used as the recipient to test the elimination frequency of the eB‐COP as previously described (Neil *et al*, 2019). Briefly, EcN KN01Δ*dapA* carrying either eB‐TP114 or eB‐TP114::Kill1 was used as donor strain to eliminate pT in EcN KN03. Conjugation experiments were carried out at 37°C for 24 h. Plasmid pT elimination was next followed on LB plates containing 1% arabinose and appropriate antibiotics by counting GFP^+^ (containing pT) and GFP‐ (pT eliminated) CFUs. Three GFP^+^ colonies from plates treated with the eB‐COP were next picked and inoculated in fresh LB broth supplemented with appropriate antibiotics. The three colonies were next used as recipients for a second round of *in vitro* conjugation.

### Mouse model

All mice‐related protocols were strictly evaluated to avoid animal suffering by the *Université de Sherbrooke Animal Care Committee*. Water and standard chow (Charles River) were provided *ad libitum* to the animals during the experiments. No more than five animals were housed in the same ventilated cage. All experiments used C57BL/6 females of 16–20 g (Charles River), which were given at least a 3‐day rest upon arrival. Daily evaluation of mice health and weight revealed no significant health or weight loss for all mice throughout the experiments.

Experiments involving *in situ* conjugation were based on a previously published model (Neil *et al*, 2020) with minor modifications. Briefly, 2 days prior to gavage (Day −2), the strain(s) used for colonization were streaked from frozen stocks onto MacConkey selective plates and incubated at 37°C overnight. The same day, 1 g/l of streptomycin was added to the mice drinking water and changed every 3 days to maintain efficacy. Adding streptomycin to the drinking water helped stabilize the colonization levels of EcN in mice as all introduced strains were resistant to streptomycin. The next day (Day −1), several colonies were inoculated in selective LB broth 5 ml pre‐cultures and incubated at 37°C. 3 to 4 h prior to mice gavage, 200–500 µl of each strain pre‐culture was transferred in 20 ml selective LB broth and allowed to grow at 37°C until an OD_600nm_ of 0.6 ± 0.1. The cells were then washed once with PBS and concentrated in a volume equivalent to 6.0 OD_600nm_ according to initial OD in LB. Each mouse then received 100 µl of the cell suspension by gavage (corresponding to ˜1 × 10^8^ CFU). Part of the inoculum was plated and analyzed for CFU counts, which ranged between 10^8^ and 10^9^ CFU/ml for all experiments except when specified otherwise in the main text. Routinely, the recipient strain was fed to mice 12 h before the donor strain unless specified otherwise. Conjugation was then monitored in feces at specified time points. For each sampling, collection tubes were prepared in advance by aseptically adding a single 0.2‐cm glass bead and 500 µl of PBS to a sterile 1.5‐ml tube. To normalize CFU by sample weight, tubes were weighted before and after sampling. Samples were next homogenized using a FastPrep‐24 (MP) instrument for 1 min at maximum speed. The samples were then serially diluted 1/10 in sterile PBS from 10^0^ to 10^−7^ of the initial concentration, and 2.5 µl of each dilution was spotted in technical duplicate on selective MacConkey plates. As a quality control for streptomycin treatment, total *Enterobacteriaceae* clearance was also followed on MacConkey plates without antibiotics for all experiments.

### Sequencing of COP escaper mutants

A total of 32 COP resistant mutants were isolated throughout the experiments shown in Figs 1 and EV1 and EV2. The genomic DNA of all donor and recipient strains used in the experiments as well as the 32 COP resistant clones was extracted. A targeted approach using multiplex PCR reactions (QIAGEN Multiplex PCR Kit) was selected to achieve high sequencing coverage in the regions of interest. Thus, the *cas9* gene, the gRNA, and the target sequence (protospacer) were distributed in 600‐bp PCR products and amplified using primers that added appropriate Illumina adaptors. A second PCR round was performed to add Illumina TruSeq barcodes for paired‐end 300 bp sequencing (Plateforme RNomics of Université de Sherbrooke). Illumina sequencing reads were then assembled using the Roche gsAssembler v2.6 software using the reference sequences for *cas9,* the gRNA(s), and the targeted *cat* gene sequence. The most likely cause of mutation was then evaluated for each escaper clone.

While most COP escapers could be fully sequenced, 7/32 clones had a gap in the *cas9* gene. Gap regions were successfully amplified by PCR and could be sequenced by Sanger sequencing. Sanger sequencing data were analyzed for any mutation that could have a detrimental effect on the activity of *cas9* (such as frameshift, non‐sense mutation, or mutation of the start codon).

### Accelerated laboratory evolution

Plasmid MP6 was transformed into EcN KN01Δ*dapA* and EcN KN01Δ*dapA* + TP114 (Appendix Table S1) by electroporation. Resulting transformants were plated on selective LB supplemented with 1% glucose to repress expression of pro‐mutagenic genes from MP6. To generate mutant libraries, EcN KN01Δ*dapA* + MP6 +TP114 was grown in LB containing 200 mM arabinose overnight at 37°C. The mutant libraries were then used as donors in a conjugation experiment for 2 h at 37°C either on agar or in broth using EcN KN03 as the recipient strain. Transconjugants were spread on at least three selective LB agar plates to keep a good diversity of mutants throughout the tests. After an overnight incubation, transconjugant cell lawns were resuspended in 9‐ml LB broth using a cell scraper and diluted 1/50 in LB containing adequate antibiotics before incubation at 37°C for 1 h. They were next used as donors to transfer TP114 mutants back to KN01Δ*dapA* + MP6 either on agar or in broth. This process was repeated for five successive rounds of mutagenesis. For each cycle, mutant libraries in EcN KN03 were cryopreserved for sequencing analyses. In addition, six isolated clones per round were kept for sequencing and further testing. For sequencing, isolated clones were inoculated in 200 µl of LB selective medium and allowed to grow overnight, while 200 µl aliquots of mutant libraries were simply thawed on ice. DNA was extracted using Quick DNA Magbead Plus Kit (Zymo Research), and DNAseq libraries were prepared using the NEBNext Ultra II FS DNA Library Prep Kit (NEB). Illumina sequencing of the mutagenesis experiments was performed at the Plateforme RNomics (https://rnomics.med.usherbrooke.ca/) of the Université de Sherbrooke on a NextSeq 500 instrument using single‐end reads of 75 bp. Processing of the reads was inspired by GenPipes (Bourgey *et al*, 2019). Reads were first trimmed based on their quality using Trimmomatic v0.32 (Bolger *et al*, 2014) with the parameters TRAILING:30 and MINLEN:30. Reads quality before and after trimming was measured with FastQC v0.11.5 (Andrew, 2010). Reads were then aligned to reference TP114 sequence from GenBank (MF521836.1) using BWA‐MEM v0.7.10 with the default parameters (Li, 2013). The aligned reads were sorted with Picard v1.123 (Available online at https://broadinstitute.github.io/picard/) and realigned with the GATK v3.7 RealignerTargetCreator and IndelRealignerSingle (Mc Kenna *et al*, 2010). Duplicates were marked with Picard v1.123 (Available online at https://broadinstitute.github.io/picard/). The haplotypes were called using GATK v3.7 Haplotype Caller and their effects were predicted using SnpEff v4.1 (Cingolani *et al*, 2012). The intergenic region between *pilV* and *rci* was removed from analysis as it contains a shufflon, for which the DNA sequence is constantly re‐arranged in the TP114 population. TP114 sequence in the GenBank record missed an A at position 52,704, which was added in the reference sequence for single nucleotide polymorphism (SNP) calling analysis. Also, TP114 gene *yadA*, *yadB*, and TP114‐054 all showed high homology to *E. coli* Nissle 1917 genome, provoking false‐positive SNP callings. These three genes were removed from heatmaps shown in the Appendix Figs S3–S5 and Fig EV3, but did not contain any enriched mutation profiles.

### 16S ribotyping

Mice feces from various treatments (PBS, EcN KN01 + TP114, COP) were sampled daily, and the DNA was purified using the ZymoBIOMICS DNA Miniprep Kit (Zymo Research) as recommended by the manufacturer. DNA was further purified using Agencourt AMPure XP DNA binding beads (Beckman Coulter) and amplified with primers binding on the V3‐V4 region of the 16S rDNA (Thijs *et al*, 2017). The PCR products were purified again and barcoded for Illumina TruSeq sequencing. The resulting data were analyzed using the Qiime2 suite v2018.8 (Bolyen *et al*, 2019) and visualized with Qiime2 view. Read counts for all detected bacterial orders in all conditions were transformed into read proportion and shown as a heatmap of its logarithmic value. Alpha diversity (Shannon) and beta diversity (weighted UniFrac) were analyzed by built‐in Qiime2 plugins.

### Generation of the CR KN04 strain

The modified CR KN04 strain was generated by Tn7 insertion of the antibiotic resistance cassettes as described previously (McKenzie *et al*, 2006) with minor adaptations. More specifically, the pGRG36‐SmCm vector (Neil *et al*, 2020) was mobilized into CR DBS100 using the RK24 conjugative machinery of the MFD*pir* ^+^ strain. To mediate cassette insertion into the terminator of *glmS*, a single colony of CR was first subcultured in broth and allowed to grow at 30°C in LB with 1% arabinose until 0.6 OD_600nm_. Then, the cultures were incubated at 42°C for 1 h before being transferred to 37°C for an overnight growth to allow for plasmid clearance. The culture was next streaked onto an LB agar plate selecting only the insert. ≥ 30 colonies were then streaked on plates containing ampicillin or streptomycin and chloramphenicol to confirm plasmid backbone elimination. Colonies that only grew in absence of ampicillin but contained the selection markers from the insert were then confirmed using primers flanking the genomic region of insertion (5′‐ GATGCTGGTGGCGAAGCTGT‐3′, 5′‐ GATGACGGTTTGTCACATGGA‐3′ and 5′‐ GAACAGGATTAGATACCCTGGTAGTCCTGCAGGCATGCCTCGAGTTGATCGGGCACGTAA‐3′).

### CR infection model

CR infection experiments followed the procedures described previously with minor adaptations (Crepin *et al*, 2016). Briefly, the CR strain was streaked from frozen stock on a MacConkey plate containing the appropriate antibiotics. The next day, 5–10 colonies were subcultured in 5 ml of LB broth and incubated overnight at 37°C. The day of the infection, 500 µl of the pre‐culture was used as an inoculum for 20 ml of LB broth. The cultures were incubated at 37°C until an OD_600nm_ of 0.6 ± 0.1. Cells were then washed once in PBS and resuspended in a volume equivalent to 6.0 OD_600nm_. Mice were orally administered 100 µl of this final mixture on day 0 of the experiment. Feces were next collected at specified timepoints and processed for CFU analysis as described in the mouse model section. Antibiotics were added in the MacConkey plates to discriminate between the *E. coli* donor (spectinomycin and kanamycin), the CR recipient KN04 (streptomycin and chloramphenicol), and the CR transconjugant KN04 (streptomycin, chloramphenicol, and kanamycin). Diarrheal scores were evaluated without knowledge of the treatments administered using a score from 0 to 4 based on the aspects of the feces (0: normal feces, 1: presence of mucus, 2: soft feces, 3: liquid feces, 4: presence of blood in the feces). For Fig 4C, streptomycin was added at 1 g/l in drinking water starting 24 h after infection and changed once every 3 days to maintain treatment efficiency up until day 8 of the experiment. When treated with bacteria (Fig 4D–F), inoculums were prepared the same way as for CR and administered to mice by gavage daily from day 1 to day 7 post‐infection.

### Statistics and reproducibility

Statistical significance was performed on the logarithmic value of the data using one‐way ANOVA unless specified otherwise. When relevant, *P*‐values are directly indicated on the graphs and represent statistical significance of the difference between the two data groups. Differences in the data were considered significant when the *P*‐value was below 0.05. Box and whiskers graphs represent the center line as the median, box encompasses the 10–90 percentiles, and whiskers are the minimal and maximal values for each data groups. All *in vitro* experiments were performed in biological triplicates using three independently grown cultures. For experiments involving mice, a minimum of four C57BL/6 female mice were used for each sampling.

## Author contributions

KN and SR designed the experiments; KN performed the experiments with assistance from PR and NA; KN analyzed the data; FG performed bioinformatic analyses for the 16S ribotyping and mutagenesis experiments; SR and VB supervised the project. KN and SR wrote the manuscript; PR, NA, FG, AM, and VB revised the manuscript.

## Conflict of interest

The work presented in the manuscript is part of patent application WO2020010452A1. All authors of the present manuscript except for P.R., A.M., and F.G. are also co‐authors of this provisional patent application. K.N. and S.R. have a financial interest in TATUM bioscience.

## Supporting information



Appendix

Expanded View Figures PDF

Dataset EV1

Source Data for Expanded View and Appendix

Source Data for Figure 1

Source Data for Figure 2

Source Data for Figure 3

Source Data for Figure 4

## Data Availability

Reads for COP escapers analysis, 16S ribotyping, and mutagenesis experiments are available at NCBI under BioProject: PRJNA510637 https://www.ncbi.nlm.nih.gov/bioproject/PRJNA510637, PRJNA510842 https://www.ncbi.nlm.nih.gov/bioproject/PRJNA510842, and PRJNA674373 https://www.ncbi.nlm.nih.gov/bioproject/PRJNA674373, respectively. Raw CFU counts and conjugation frequency evaluation are available in the source data file. The sequences of plasmids used in this study were deposited on GenBank. Accession numbers are listed in Appendix Table S1.
