# Supplementary material for: High‐efficiency delivery of CRISPR‐Cas9 by engineered probiotics enables precise microbiome editing
Source: Mol Syst Biol. 2021 Oct 19;17(10):e10335. doi: 10.15252/msb.202110335 (PMC8527022; doi:10.15252/msb.202110335)
Supplement: Supplementary file 1 — Appendix [file MSB-17-e10335-s008.pdf]

Appendix

High-efficiency Delivery of CRISPR-Cas9 by engineered probiotics enables precise microbiome editing

Authors: Kevin Neil[1], Nancy Allard[1§], Patricia Roy[1§], Frédéric Grenier[1], Alfredo Menendez[2], Vincent Burrus[1], and Sébastien Rodrigue[1].

Affiliations:

[1] Département de biologie, Université de Sherbrooke, Sherbrooke, Québec, Canada, J1K 2R1.

[2] Département de microbiologie et infectiologie, Université de Sherbrooke, Sherbrooke, Québec, Canada, J1E 4K8.

§ These authors contributed equally to this work.

*Correspondence to: Sebastien.Rodrigue@USherbrooke.ca

This Appendix includes:

Appendix Figures S1-S6

Appendix Table S1

Appendix References

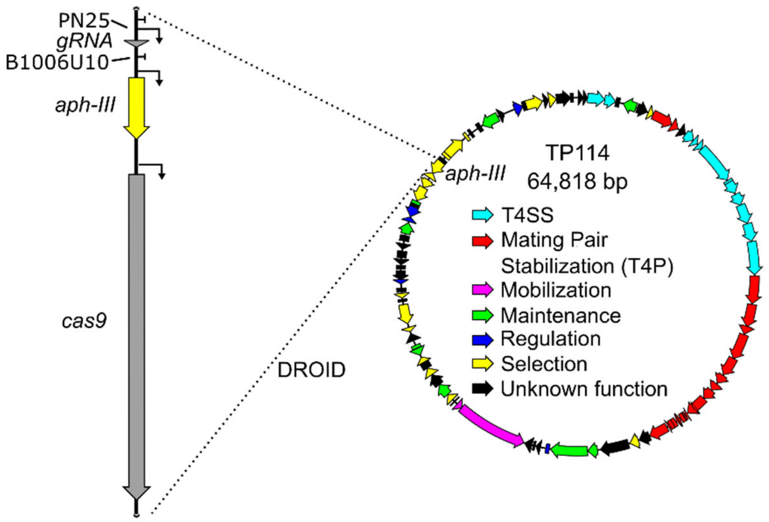

Appendix Figure S1. Map of the COP genetic construct.

Schematic representation of the Kill module insertion in TP114 by DROID (Neil *et al*, 2019), which replaced the endogenous *aph-III* gene. The general function of the genes on TP114 is color coded.

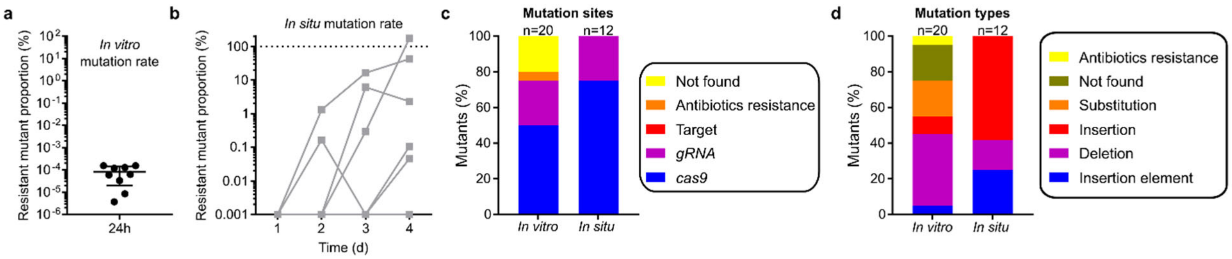

Appendix Figure S2. Analysis of COP treatment escapers.

a Frequency of transconjugants (escapers) following a 2-hour conjugation assay in broth between the COP strain and the target bacteria KN02 (n=9). Line and error bars represent the average and standard deviation of the data respectively.

b Proportion of the KN02 target bacterium population from Figure 1h that acquired TP114::Kill1 but survived (n=8). Escapers from both *in vitro* and *in situ* experiments were sequenced and the integrity of the CRISPR-*cas9* module and targeted *cat* gene sequences were analyzed. The dotted line highlights the mutant proportion at which the target bacteria population can be considered to be composed entirely of escaper mutants.

c-d Localization of deleterious mutations (**c**) and mutation types (**d**) for escapers recovered in both conditions.

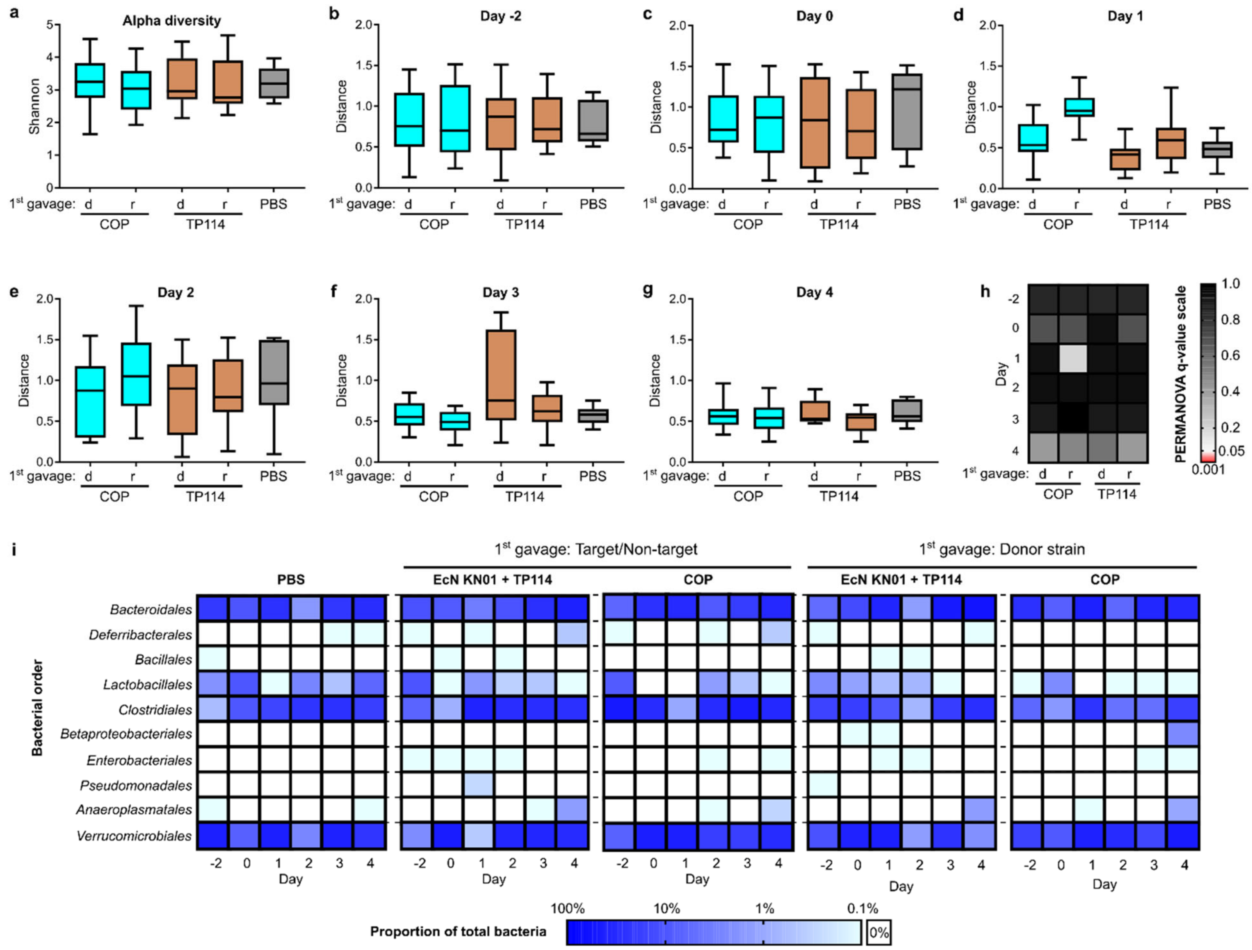

Appendix Figure S3. COP treatment has no major effect on microbiota composition.

a-h Microbiota diversity for each treatment groups (see Figure 1 and Figure EV2) from day 1 to 4 evaluated using Shannon's index (**a**) or beta diversity calculated by weighted unifrac comparing each group to the PBS control at day -2 (**b**), 0 (**c**), 1 (**d**), 2 (**e**), 3 (**f**), and 4 (**g**). The corrected significance of beta diversity divergence to the PBS control is shown as a heatmap (**h**). Days are given relative to the first gavage on day 0. Day -2 represents the beginning of the streptomycin treatment. Mice groups are divided by treatment types (COP, TP114 or PBS) and strains used for the first gavage, i.e. donor (d), recipient (r) or phosphate buffered saline (PBS). All groups contained at least 4 mice. For all box and whiskers plots, medians are shown as a bar, boxes represent the 10-90% percentile of the data and whiskers extend to the minimal and maximal values.

i Relative abundance of 10 bacterial orders by 16S ribotyping in mice groups treated with PBS, EcN KN01 + TP114 or the COP (n=4).

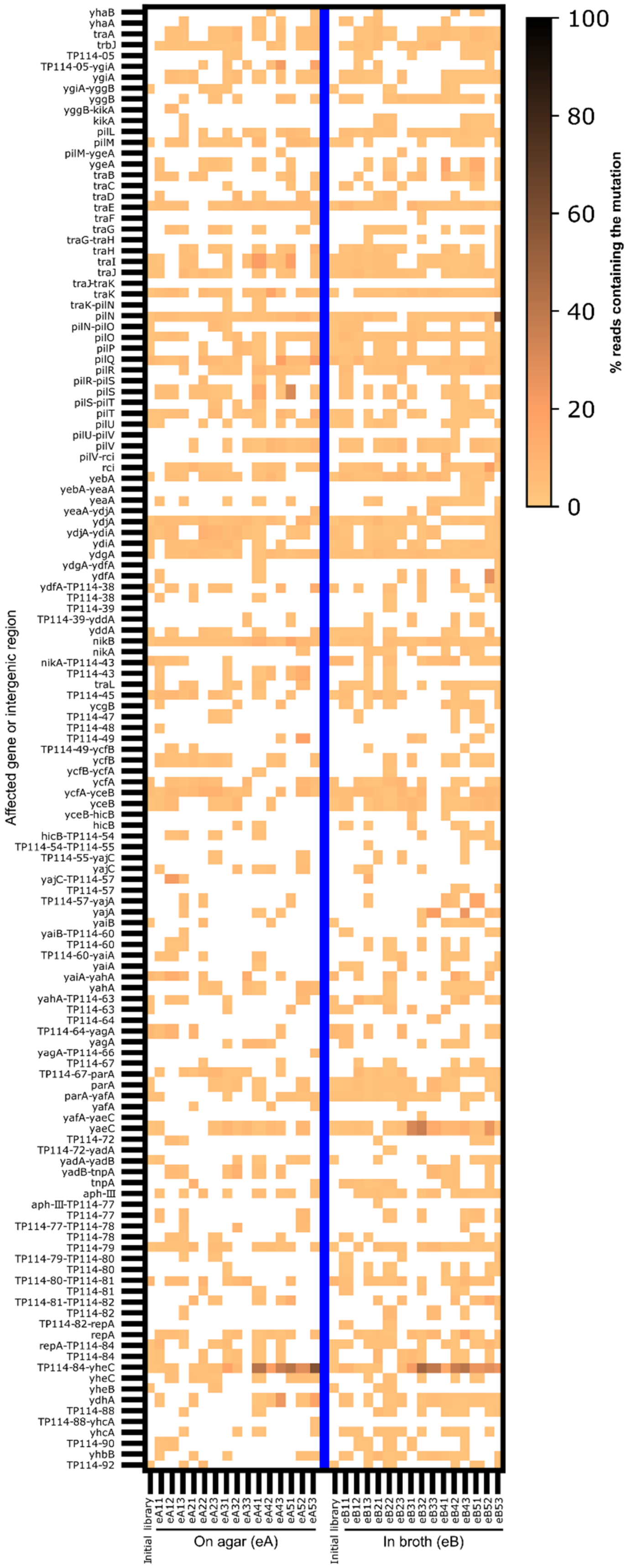

Affected gene or intergenic region
yhaB
yhaA
traA
trbJ
TP114-05
TP114-05-ygiA
ygiA
ygiA-yggB
yggB
yggB-kikA
kikA
pilL
pilM
pilM-ygeA
ygeA
traB
traC
traD
traE
traF
traG
traG-traH
traH
traI
traJ
traJ-traK
traK
traK-pilN
pilN
pilN-pilO
pilO
pilP
pilQ
pilR
pilR-pilS
pilS
pilS-pilT
pilT
pilU
pilU-pilV
pilV
pilV-rci
rci
yebA
yebA-yeaA
yeaA
yeaA-ydjA
ydjA
ydjA-ydiA
ydiA
ydgA
ydgA-ydfA
ydfA
ydfA-TP114-38
TP114-38
TP114-39
TP114-39-yddA
yddA
nikB
nikA
nikA-TP114-43
TP114-43
traL
TP114-45
ycgB
TP114-47
TP114-48
TP114-49
TP114-49-ycfB
ycfB
ycfB-ycfA
ycfA
ycfA-yceB
yceB
yceB-hicB
hicB
hicB-TP114-54
TP114-54-TP114-55
TP114-55-yajC
yajC
yajC-TP114-57
TP114-57
TP114-57-yajA
yajA
yaiB
yaiB-TP114-60
TP114-60
TP114-60-yaiA
yaiA
yaiA-yahA
yahA
yahA-TP114-63
TP114-63
TP114-64
TP114-64-yagA
yagA
yagA-TP114-66
TP114-67
TP114-67-parA
parA
parA-yafA
yafA
yafA-yaeC
yaeC
TP114-72
TP114-72-yadA
yadA-yadB
yadB-tnpA
tnpA
aph-III
aph-III-TP114-77
TP114-77
TP114-77-TP114-78
TP114-78
TP114-79
TP114-79-TP114-80
TP114-80
TP114-80-TP114-81
TP114-81
TP114-81-TP114-82
TP114-82
TP114-82-repA
repA
repA-TP114-84
TP114-84
TP114-84-yheC
yheC
yheB
ydhA
TP114-88
TP114-88-yhcA
yhcA
TP114-90
yhbB
TP114-92
Initial library
eA11
eA12
eA13
eA21
eA22
eA23
eA31
eA32
eA33
eA41
eA42
eA43
eA51
eA52
eA53
On agar (eA)
Initial library
eB11
eB12
eB13
eB21
eB22
eB23
eB31
eB32
eB33
eB41
eB42
eB43
eB51
eB52
eB53
In broth (eB)
100
80
60
40
20
0
% reads containing the mutation

Appendix Figure S4. Accelerated laboratory evolution selected for mutations in specific regions of TP114.

Mutation heatmap showing the proportion of reads detecting a mutation in a specific gene or intergenic region in mutant libraries selected for conjugation on agar or in broth. Mutant libraries are named as either evolved on agar (eA) or in broth (eB), followed by two digits that identify the mutagenesis round and the library replicate, respectively (resulting in an eAXX or eBXX identifier). The blue line defines the limit between mutant libraires evolved on agar or in broth.

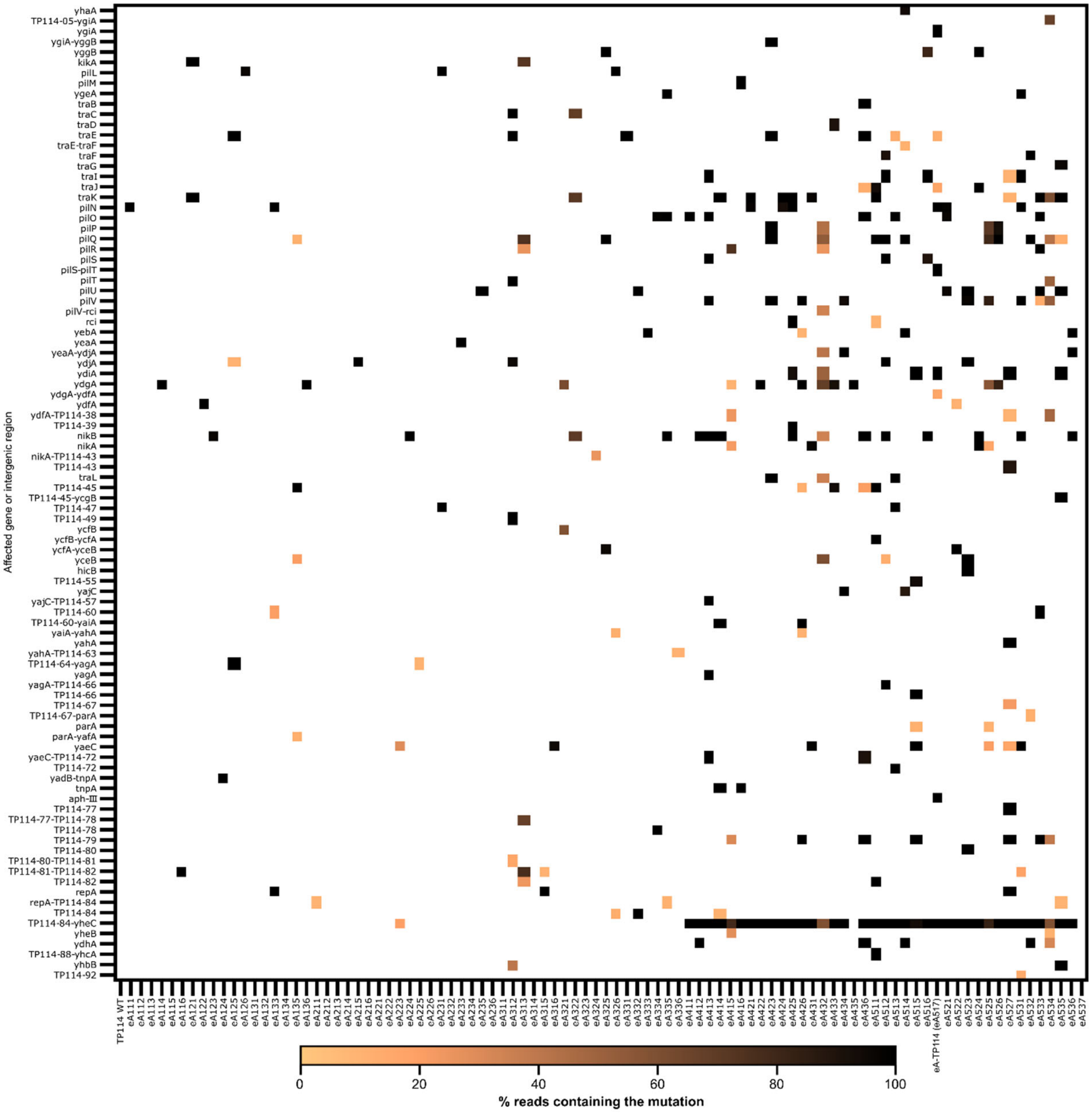

Appendix Figure S5. Isolated clones from mutagenesis rounds on agar present enriched mutations in specific regions of TP114.

Mutation heatmap showing the proportion of reads detecting a sequence variant in a specific gene or intergenic region in isolated clones. The clones obtained from evolution experiments selected on agar were named with the letters eA, followed by a three-digit suffix respectively corresponding to the mutagenesis round, the replicate, and the clone number (eAXXX).

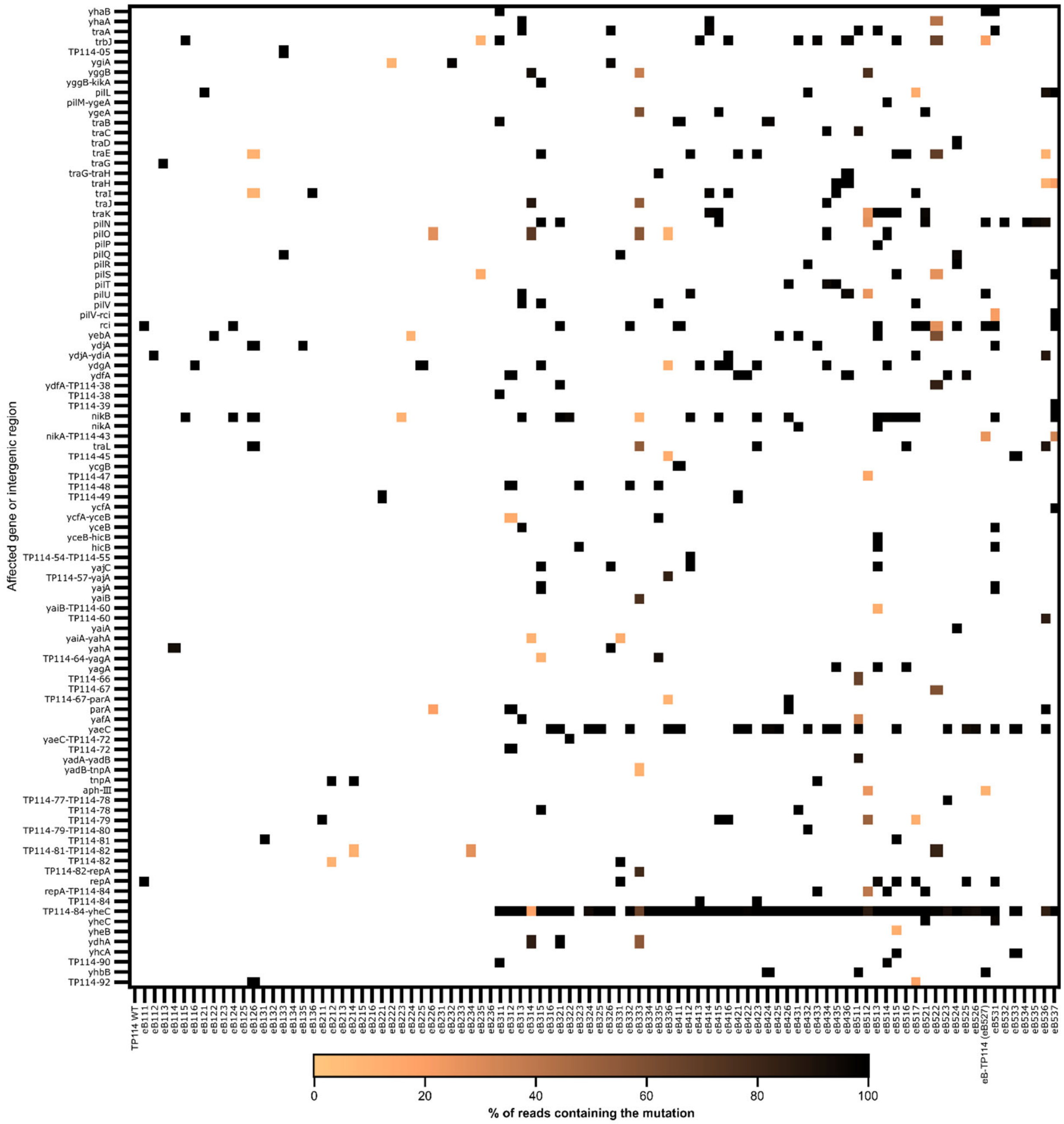

Appendix Figure S6. Isolated clones from mutagenesis rounds in broth present enriched mutations in specific regions of TP114.

Mutation heatmap showing the proportion of reads detecting a sequence variant in a specific gene or intergenic region in isolated clones. The clones obtained from evolution experiments selected in broth were named with the letters eB, followed by a three-digit suffix respectively corresponding to the mutagenesis round, the replicate, and the clone number (eBXXX).

Appendix Table S1: List of strains and plasmids used in this study.

Strain or plasmid	Relevant phenotype or genotype*	Source/Reference
Escherichia coli		
AACSI01	Swine feces isolate serotype O25:H2, carries 4 natural plasmids	Kind gift from Dominic Poulin-Laprade. Cefotaxime resistant *E. coli* isolates from swine feces. Isolated from Canadian farms by Agriculture and Agri-food Canada
AACSI02	Swine feces isolate serotype O58:H21, carries 5 natural plasmids	
AACSI03	Swine feces isolate serotype O76:H7, carries 8 natural plasmids	
AACSI04	Swine feces isolate serotype O24:H4, carries 7 natural plasmids	
CP9	Extraintestinal pathogenic *E. coli*, isolated from a bacteriemia, O4:K54:H5	Kind gift from Pr. Charles Dozois (Russo & Singh, 1993)
J96	Uropathogenic *E. coli*, O4:K6, isolated from a pyelonephritis patient	Kind gift from Pr. Charles Dozois (Klein & Gitai, 2013)
MT78	Avian pathogenic *E. coli*, O2:H^+	Kind gift from Pr. Charles Dozois (Matter et al., 2011)
RS218	Neo-natal meningitis causing *E. coli*, O18:H7:K1	Kind gift from Pr. Charles Dozois (Wijetunge *et al.*, 2015)
KN01	Sm^r, Sp^r Nissle 1917	(Neil *et al.*, 2020)
KN01Δ*dapA*	Δ*dapA* KN01	
KN02	Sm^r, Cm^r Nissle 1917	
KN03	Sm^r, Tc^r Nissle 1917	
COP	KN01 + TP114::Kill1	This study
eB-COP	KN01 + eB-TP114::Kill1	This study
EC100D*pir*+	F– *mcrA* Δ(*mrr-hsdRMS-mcrBC*) ϕ80d*lacZ*ΔM15 Δ*lacX74 recA1 endA1 araD*139 Δ(ara, leu)7697 *galU galK* λ– *rpsL nupG pir*+ (DHFR)	#ECP09500 (Lucigen)
MFD*pir*+	Δ*dapA*, RP4 mobilization machinery inserted in chromosome	(Ferrières *et al*., 2010)
MG1655Nx^R	K-12 *F– λ– ilvG– rfb-50 rph-1* Nx^r	(Carraro *et al*., 2014)
Nissle 1917	Wildtype probiotic strain	DSM-6601 (DSMZ)
Citrobacter rodentium		
DBS100	Model strain closely related to human EPEC and EHEC strains	Kind gift from Pr. Alfredo Menendez (Popov *et al*., 2019)
KN04	Sm^R, Cm^R DBS100 derivative	This study
**Conjugative plasmids**		
TP114	$IncI_2$, Km^r	DSM-4246 (DSMZ) , Genbank accession: MF521836.1
eA-TP114 (eA517)	Evolved by accelerated laboratory evolution for 5 cycles on agar	This study
eB-TP114 (eB527)	Evolved by accelerated laboratory evolution for 5 cycles in broth	This study
**Plasmids**		
pBXB1	$oriV_{pMB1}$, *bxb1* integrase, *bla* (Ap^r)	Genbank: MK756311(Neil *et al*., 2019)
pE-FLP	$oriV_{pSC101ts}$, *flp*, Ap^r	Addgene #45978
pGRG36-SmCm	Tn7 machinery to insert the *aad7* and *cat* genes in the *glmS* terminator region	(Neil *et al.* 2020, McKenzie *et al*., 2006)
pKill1	$oriV_{pSC101ts}$, $attP_{bxb1}$, FRT, 1 gRNA vs *cat*, *aph-IIIa* (Km^r), *cas9*	Genbank: MK756312(Neil *et al*., 2019)
MP6	Mutagenesis plasmid	(Badran & Liu, 2015)
pREC1	$oriV_{R6K}$, $attB_{bxb1}$, *tetB* (Tc^r), FRT	Genbank: MK756315(Neil *et al*., 2019)
pSIM6	$oriV_{pSC101ts}$, Lambda Red recombinase, Ap^r	(Datsenko & Wanner, 2000)
pT	$oriV_{pMB1}$, *sfGFP*, *araC*, *cat*.	IGEM : BBa_I746908 (Neil *et al*., 2019)
TP114::Kill1	TP114::*tetB*-Kill1 after FRT driven deletion of *tetB* and $oriV_{pSC101ts}$	(Neil *et al*., 2019)
TP114::*tetB*	TP114Δ*aph-III*::*tetB*	(Neil *et al*., 2019)
TP114::*tetB*-Kill1	TP114::*tetB* with inserted Killing module 1	(Neil *et al*., 2019)
eB-TP114::Kill1	eB-TP114::*tetB*-Kill1 after FRT driven deletion of *tetB* and $oriV_{pSC101ts}$	This study
eB-TP114::*tetB*	eB-TP114Δ*aph-III*::*tetB*	This study
eB-TP114::*tetB*-Kill1	eB-TP114::*tetB* with inserted Killing module 1	This study

*Antibiotic resistance: Ap^r: Ampicillin, Cm^r: Chloramphenicol, Km^r: Kanamycin, Nx^r: Nalidixic acid, Sp^r: Spectinomycin, Sm^r: Streptomycin, Su^r: Sulfamethoxazole, Tc^r: Tetracycline, and Tm^r: Trimethoprim.

Appendix References

Badran, A. H., & Liu, D. R. (2015). Development of potent in vivo mutagenesis plasmids with broad mutational spectra. *Nature Communications*, *6*(8425), 1–10. https://doi.org/10.1038/ncomms9425

Carraro, N., Matteau, D., Luo, P., & Burrus, V. (2014). The Master Activator of IncA / C Conjugative Plasmids Stimulates Genomic Islands and Multidrug Resistance Dissemination. *PLoS Genetics*, *10*(10), e1004714. https://doi.org/10.1371/journal.pgen.1004714

Datsenko, K. A., & Wanner, B. L. (2000). One-step inactivation of chromosomal genes in *Escherichia coli* K-12 using PCR products. *PNAS*, *97*(12), 6640–6645.

Ferrières, L., Hémery, G., Nham, T., Guérout, A. M., Mazel, D., Beloin, C., & Ghigo, J. M. (2010). Silent mischief: Bacteriophage Mu insertions contaminate products of *Escherichia coli* random mutagenesis performed using suicidal transposon delivery plasmids mobilized by broad-host-range RP4 conjugative machinery. *Journal of Bacteriology*, *192*(24), 6418–6427. https://doi.org/10.1128/JB.00621-10

Klein, E. A., & Gitai, Z. (2013). Draft genome sequence of uropathogenic *Escherichia coli* strain J96. *Genome Announcements*, *1*(1), 6–7. https://doi.org/10.1128/genomeA.00245-12

Matter, L. B., Barbieri, N. L., Nordhoff, M., Ewers, C., & Horn, F. (2011). Avian pathogenic *Escherichia coli* MT78 invades chicken fibroblasts. *Veterinary Microbiology*, *148*(1), 51–59. https://doi.org/10.1016/j.vetmic.2010.08.006

McKenzie, G. J., Craig, N. L., Grozdanov, L., Zähringer, U., Blum-Oehler, G., Brade, L., Henne, A., Knirel, Y., Schombel, U., Schulze, J., Sonnenborn, U., Gottschalk, G., Hacker, J., Rietschel, E., Dobrindt, U., Bumann, D., Hueck, C., Aebischer, T., Meyer, T., … Maniatis, T. (2006). Fast, easy and efficient: site-specific insertion of transgenes into Enterobacterial chromosomes using Tn 7 without need for selection of the insertion event. *BMC Microbiology*, *6*(39), 1–7. https://doi.org/10.1186/1471-2180-6-39

Neil, K., Allard, N., Grenier, F., Burrus, V., & Rodrigue, S. (2020). Highly efficient gene transfer in the mouse gut microbiota is enabled by the Incl2 conjugative plasmid TP114. *Communications Biology*, *3*(523), 1–9. https://doi.org/10.1038/s42003-020-01253-0

Neil, K., Allard, N., Jordan, D., & Rodrigue, S. (2019). Assembly of large mobilizable genetic cargo by double recombinase operated insertion of DNA (DROID). *Plasmid*, *104*(June),

102419. https://doi.org/10.1016/j.plasmid.2019.102419

Popov, G., Fiebig-Comyn, A., Shideler, S., Coombes, B. K., & Savchenko, A. (2019). Complete Genome Sequence of Citrobacter rodentium Strain DBS100. *Microbiology Resource Announcements*, *8*(24), 1–2. https://doi.org/10.1128/mra.00421-19

Russo, T. A., & Singh, G. (1993). An extraintestinal, pathogenic isolate of *Escherichia coli* (O4/K54/H5) can produce a group 1 capsule which is divergently regulated from its constitutively produced group 2, K54 capsular polysaccharide. *Journal of Bacteriology*, *175*(23), 7617–7623. https://doi.org/10.1128/jb.175.23.7617-7623.1993

Wijetunge, D. S. S., Katani, R., Kapur, V., & Kariyawasama, S. (2015). Complete genome sequence of *Escherichia coli* strain RS218 (O18: H7: K1), associated with neonatal meningitis. *Genome Announcements*, *3*(4), 5–6. https://doi.org/10.1128/genomeA.00804-15
