# Supplementary material for: High‐efficiency delivery of CRISPR‐Cas9 by engineered probiotics enables precise microbiome editing
Source: Mol Syst Biol. 2021 Oct 19;17(10):e10335. doi: 10.15252/msb.202110335 (PMC8527022; doi:10.15252/msb.202110335)
Supplement: Supplementary file 2 — Expanded View Figures PDF [file MSB-17-e10335-s003.pdf]

Expanded View Figures

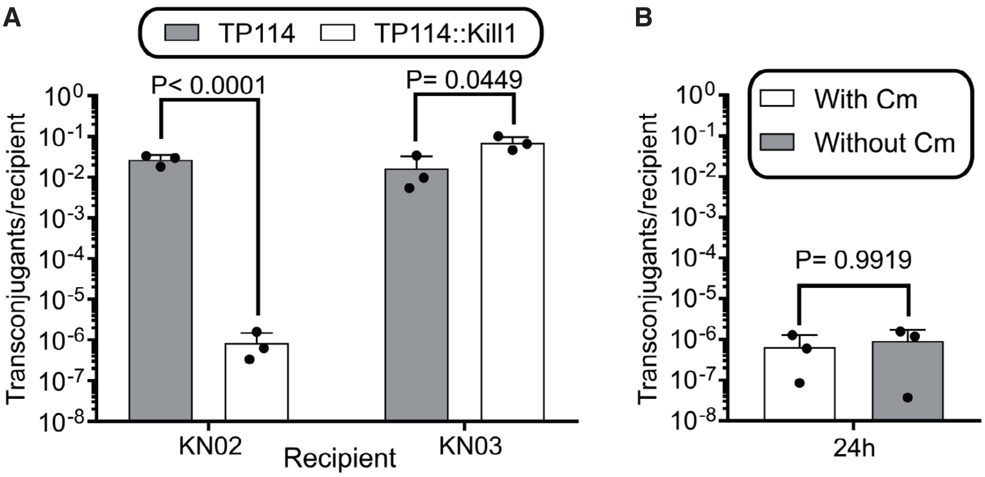

Figure EV1. COPs can selectively eliminate a target strain from a mixed population *in vitro*.

A Transfer frequency of TP114::Kill1 and TP114 from KN01Δ*dapA* toward a 1:1 mix of target (KN02) and non-target (KN03) recipient bacteria after 24 h.

B Selection of TP114::Kill1 transconjugants with or without chloramphenicol (Cm) allows the distinction between KN02 killing and Cm sensitization.

Data information: Bars and error bars, respectively, show the mean and standard deviation of the mean of three biological replicates (n = 3). Statistical significance was determined using a one-way ANOVA test on the log of the data.
Source data are available online for this figure.

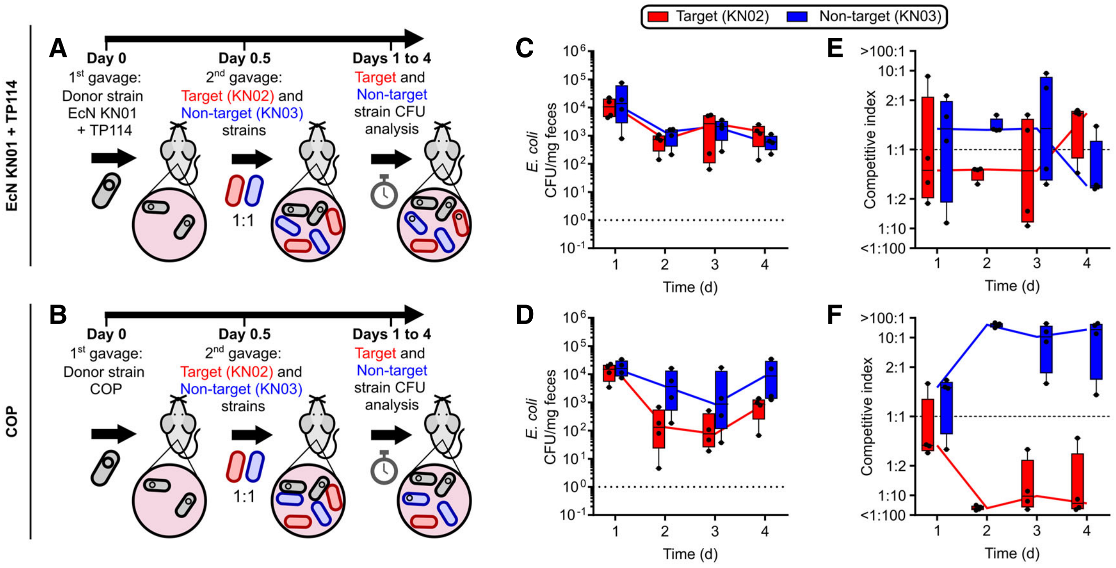

Figure EV2. COPs can protect the microbiota from colonization of a specific strain.

A, B Mouse model experiment. Mice either received a dose of an EcN KN01 + TP114 (A) or the COP (B) 12 h prior to the introduction of the target/non-target 1:1 mix.

C, D CFU abundance of the target and non-target strains for the mice groups given EcN KN01 + TP114 (C) or the COP (D) followed in feces for up to 4 days post-gavage (n = 4). The dotted line indicates the highest detection limit based on feces weight (10^0).

E, F Competitive indexes calculated from the CFU ratio between both strains in mice treated prophylactically with either EcN KN01 + TP114 (e) or the COP (f) (n = 4). The dotted line highlights the expected competitive index at equilibrium.

Data information: (C–F) For all box and whiskers plots, medians are shown as a bar, boxes represent the 10–90% percentile of the data, and whiskers extend to the minimal and maximal values.
Source data are available online for this figure.

Figure EV3. Correlation between mutation location and transfer rates of TP114 evolved clones.

A Transfer rates of wild-type TP114 in broth compared to 30 clones of TP114 evolved for conjugation in broth (eB) and isolated at different mutagenesis cycles (n = 3, biological triplicate). The transfer rate of each clone is shown above the heatmap representing its mutation profile, with the color scale representing the proportion of reads confirming the presence of a variant. Bar and error bars represent the average and standard deviation of the data. The blue bar highlights the conjugation rates of the eB-TP114 selected for the eB-COP system (eB527).

B Mutation positions and associated transfer rates for the intergenic region located between TP114-084 and TP114-085. Transfer rates of clones for which no mutation was detected in this region were grouped under "no mutation". The blue tick marks on the x-axis indicate the position of mutations found in the intergenic region between TP114-084 and TP114-085 in all clones presented in Appendix Fig S6. Clones are named as described in Appendix Fig S6. Error bars represent the standard deviation of the data points.

Source data are available online for this figure.

 © 2021 The Authors

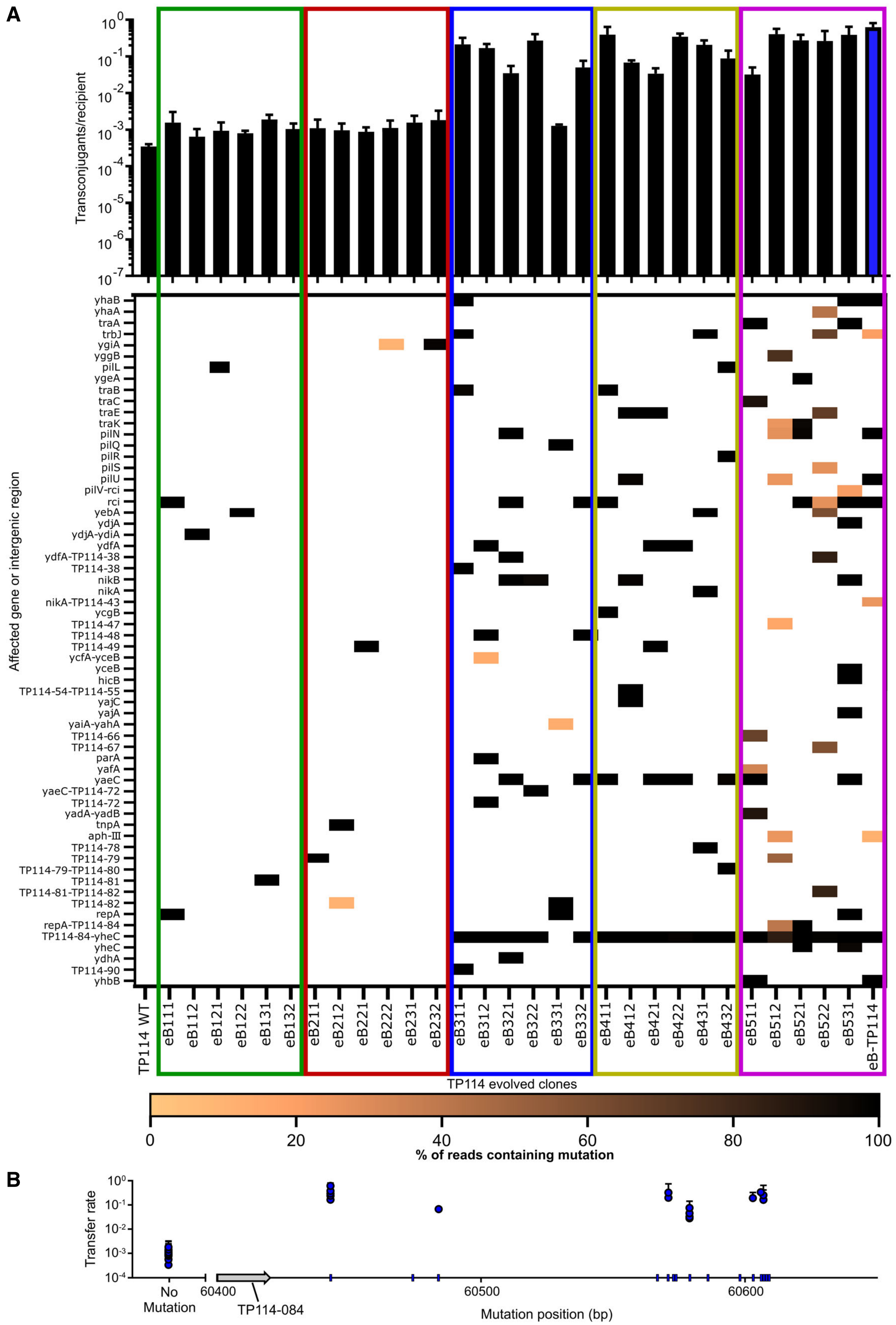
A
Transconjugants/recipient
Affected gene or intergenic region
yhaB
yhaA
traA
trbJ
ygiA
yggB
pilL
ygeA
traB
traC
traE
traK
pilN
pilQ
pilR
pilS
pilU
pilV-rci
rci
yebA
ydjA
ydjA-ydiA
ydfA
ydfA-TP114-38
TP114-38
nikB
nikA
nikA-TP114-43
ycgB
TP114-47
TP114-48
TP114-49
ycfA-yceB
yceB
hicB
TP114-54-TP114-55
yajC
yajA
yaiA-yahA
TP114-66
TP114-67
parA
yafA
yaeC
yaeC-TP114-72
TP114-72
yadA-yadB
tnpA
aph-III
TP114-78
TP114-79
TP114-79-TP114-80
TP114-81
TP114-81-TP114-82
TP114-82
repA
repA-TP114-84
TP114-84-yheC
yheC
ydhA
TP114-90
yhbB
TP114 WT
eB111
eB112
eB121
eB122
eB131
eB132
eB211
eB212
eB221
eB222
eB231
eB232
eB311
eB312
eB321
eB322
eB331
eB332
eB411
eB412
eB421
eB422
eB431
eB432
eB511
eB512
eB521
eB522
eB531
eB-TP114
TP114 evolved clones
% of reads containing mutation
0
20
40
60
80
100
B
Transfer rate
No Mutation
60400
TP114-084
60500
60600
Mutation position (bp)

Figure EV3.

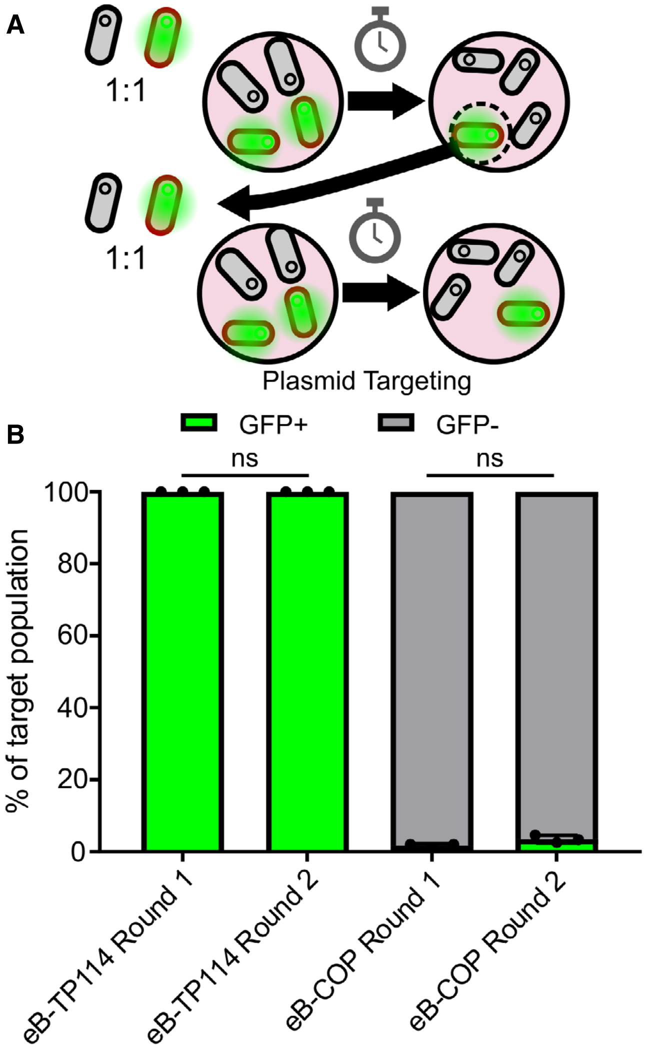

Figure EV4. The remaining fraction of target bacteria after eB-COP remains susceptible to the system.

A Overview of the re-exposition experiment. A recipient strain carrying a GFP plasmid encoding the targeted *cat* gene was mixed with the eB-COP strain. After a first round of plasmid elimination, three residual GFP-positive colonies were selected for a re-challenge with a second round of eB-COP.

B Plasmid elimination measured by counting the proportion of GFP positive (GFP^+) and GFP negative (GFP^-) colonies after one or two successive rounds of COP treatment. As a control, the same strain was exposed to KN01 carrying eB-TP114 without a CRISPR module. All experiments were performed in biological triplicates (n = 3), and error bars represent the standard deviation of the data points.

Source data are available online for this figure.

 © 2021 The Authors
