# Supplementary material for: High‐efficiency delivery of CRISPR‐Cas9 by engineered probiotics enables precise microbiome editing
Source: Mol Syst Biol. 2021 Oct 19;17(10):e10335. doi: 10.15252/msb.202110335 (PMC8527022; doi:10.15252/msb.202110335)
Supplement: Supplementary file 9 [file MSB-17-e10335-s006.pdf]

molecular systems biology

High-efficiency Delivery of CRISPR-Cas9 by engineered probiotics enables precise microbiome editing

Kevin Neil, Nancy Allard, Patricia Roy, Frédéric Grenier, Alfredo Menendez, Vincent Burrus, and Sébastien Rodrigue
DOI: 10.15252/msb.202110335

Corresponding author(s): Sébastien Rodrigue (Sebastien.Rodrigue@USherbrooke.ca)

Review Timeline:

Submission Date:	9th Mar 21
Editorial Decision:	6th Apr 21
Revision Received:	12th Jul 21
Editorial Decision:	13th Aug 21
Revision Received:	9th Sep 21
Accepted:	13th Sep 21

Editor: Jingyi Hou

Transaction Report:

(Note: With the exception of the correction of typographical or spelling errors that could be a source of ambiguity, letters and reports are not edited. Depending on transfer agreements, referee reports obtained elsewhere may or may not be included in this compilation. Referee reports are anonymous unless the Referee chooses to sign their reports.)

1st Editorial Decision 6th Apr 2021

Thank you for submitting your work to Molecular Systems Biology. We have now heard back from the three reviewers who agreed to evaluate your manuscript. As you will see from the reports below, the reviewers acknowledge the potential interest of the study. They raise however a series of concerns, which we would ask you to address in a major revision.

Since the reviewers' recommendations are rather clear, there is no need to reiterate all the points listed below. All issues raised by the reviewers need to be satisfactorily addressed. As you may already know, our editorial policy allows in principle a single round of major revision and it is therefore essential to provide responses to the reviewers' comments that are as complete as possible.

On a more editorial level, we would ask you to address the following issues.

REFEREE REPORTS

Reviewer #1:

Neil et al. report the engineering of a probiotic strain capable of transferring CRISPR-cas9 machinery to different bacterial species related to E. coli in the gastrointestinal tract. They achieve this by employing a conjugative plasmid previously identified by the authors in a different study and evolving this system to make it capable of high-efficiency DNA transfer. The authors then introduced this system into a probiotic E. coli strain. They used it to eliminate antibiotic-resistant E. coli and clear a C. rodentium infection, both in the mouse gut. This probiotic treatment approach does not seem to disturb the rest of the microbiota and has efficiencies comparable to

conventional antibiotics in mice.

This study is an exciting demonstration of how molecular tools and systems biology methods can be employed to tackle the antibiotic resistance crisis. This study should be relevant to researchers in translational systems and synthetic biology. The conclusions are well supported by the experiments and the data presented.

Major comments:
1. The authors do a great job explaining the way they engineer strain discrimination in simulated infection scenarios. However, they have not described how the CRISPR-cas9 kills the bacteria.
o The authors should elaborate on the precise killing mechanism triggered by the gene-editing machinery targeting antibiotic resistance genes. The manuscript focuses more on explaining that the gene transference occurs and that the target strain is eliminated. But there is no mention of the specific killing mechanism potentially caused by double-strand breaks in the target gene.
o How do the authors differentiate elimination of the antibiotic-resistant strain vs. elimination of specific genes? Because the assessment of the clearance is typically based on CFU counting in selective agar, is it possible that the CRISPR-Cas only caused a mutation in the selection gene but did not kill the bacteria? The different selection mechanisms used to count CFU are also difficult to follow, making it challenging to find a clear answer to the above question.
o If the antibiotic-resistance gene is located on a plasmid, will the CRISPR-Cas-based killing strategy still work? Are plasmid- or chromosome-based resistance genes more common in clinical setting? The authors should discuss any limitation of the CRISPR-Cas strategy to target natural pathogenic bacteria.

2. Line 107-110. The language lacks precision. For instance, "remained similar between the controls and the COP" is unclear. The data is rather complex, and it is unclear how "similarity" is quantified or justified. Can the authors support their statement using quantitative metrics? The analysis is critical because the authors repeatedly claim that their method "leaves the rest of the microbiota essentially untouched".

Minor comments:
• The authors should discuss possible clearance strategies for the COP strains after the infection has been eliminated. In biosafety consideration, isn't a genetically modified bacterial species typically disabled from conjugation to prevent any horizontal gene transfer? Is the authors' strategy consistent with the current safety regulation? If not, the authors should discuss future challenges of their strategy in term of biosafety and clinical application.

- The labeling of the Nissle strains (KN01, KN02, KN03, EcN KN01 + TP114, etc.) is confusing. The authors might want to rename these strains with more informative names to prevent the reader from going back and forth between the main text and the supplementary material to find the information on the genotype and phenotype. It will also be great to include more descriptive information in the figure labels.

- Why is there a difference in the target/non-target ratio between Figs 1 and 3 (1:1 and 1000:1, respectively)? If the authors are trying to demonstrate that the eB-COP strain performs better than the COP strain, shouldn't the ratios be kept the same?

Reviewer #2:

I review this article non-anonymously, Christian Lesterlin Cell-to-Cell DNA transfer lab (CNRS, Molecular microbiology and Structural biochemistry).

In the manuscript "High-efficiency Delivery of CRISPR-Cas9 by engineered probiotics enables precise microbiome editing", Neil and co-workers investigate the possibility of using conjugation plasmids to deliver CRISPR systems that exert antibacterial activity. This promising strategy has been the focus of several recent publications in the past few years to target E. coli (Citorik et al., 2014; Dong et al., 2019; Ruotsalainen et al., 2019; Wang et al., 2019), Salmonella Typhimurium (Hamilton et al., 2019) or Enterococcus faecalis (Rodrigues et al., 2019). The authors should also cite and discuss the recent article by Reuter and co-workers (Reuter et al., 2021), which probably came out after the finalization of the present manuscript.
There are several bottlenecks to overcome to validate this type of approach as a practical non-antibiotic antibacterial approach for in situ microbiota manipulation/editing. One major limitation is the efficiency of plasmids transfer in situ. In this work, the authors specifically tackle this question by performing Accelerated Laboratory Evolution (ALE), resulting in the successful isolation of TP114 mutants that exhibit enhanced transfer rates. In the gut, the eB-TP114 clone (eB527) exhibits improved transfer compared to the native TP114. The conjugation system of the eB-TP114 has then been chosen to enhance the efficiency of the COP system in further mouse gut experiments. Remarkably, the authors convincedly show that the eB-COP plasmid allows the elimination of drug-resistant Escherichia coli and the mouse pathogen Citrobacter rodentium in the mouse gut.
The manuscript is concise and well written, the experiments are performed to a high standard, and the results are well presented in the figures. This is a very nice manuscript and the presented conclusions follow from careful analysis of the data. As it is, the manuscript is of appropriate quality and significance for publication. Nonetheless, I propose to consider some minor changes and I suggest/recommend only one additional experiment, which would be required to ascertain the observation regarding the absence of escaper mutants (see last point below).

Specific points:
I think it would be very helpful to add the genetic map of the killer plasmid to the main figure 1, for the reader to grasp the COP system in one glance.

Identification of TP114 "superspreadrer" variants is a most significant achievement. It provides a conjugation system that performs high transfer efficiency in the mouse gut, at least between E. coli donors and E. coli/C rodentium recipients. TP114 variants carry mutations in the TP114-084/TP114-085 intergenic region. The eB-TP114 mutants were additionally enriched with mutations in a predicted transcription regulator yaeC, whereas "eA-TP114 mutants displayed an increased number of deleterious mutations in the pil genes compared to eB-TP114". In the gut, the eB-TP114 clone (eB527) (but not the eA-TP114 clone (eA517)) exhibits improved transfer compared to TP114.
I agree that, as stated by the authors (line 259): "The implication of these genes for TP114 conjugation will require more investigation". Nonetheless, it would be interesting for the reader to have more details regarding the author's interpretation of the predicted/hypothetical impact of these mutations of the conjugation rates (possible role of yaeC mutations and other mutations). The authors should then consider elaborating a bit more on this point in the discussion.
In the same idea, I think that the statement (line 275): "The eB-COP with its constitutively active transfer machinery would have a more decisive impact on the infection." should be rephrases. It is likely indeed that the expression of the conjugation machinery is affected (upregulated), yet at this stage, this is not the only possible explanation for the observed enhance transfer of the mutated TP114.

Since several antibacterial systems based on conjugation and CRISPR have been published, the authors should discuss and emphasize their methodology's pros and cons compared to previous reports. In particular, they should explain the choice of using autonomous conjugative plasmids instead of smaller mobilizable plasmids, which can be transferred using the conjugation machinery encoded in trans on an additional helper plasmid on the chromosome of the donor strain. It could also be appreciated to have a few words on the biocontainment aspects regarding the uncontrolled propagation of such antibacterial plasmids that could be used in therapeutic treatments.

While developing tools for microbiome editing, it is critically important to ensure that the CRISPR systems will be active in the target strain only and have no impact on the other species present in the microbiota). To that end, the authors use metagenomic analysis of the microbiota composition and show that COP had no significant effect on the microbiota diversity. Yet, in the experiments presented, mice were initially treated with Streptomycin (1g/L), which results in the "simplification" of the composition of their microbiota in terms of microbial diversity. To achieve strain-specific targeting in more complex natural microbiotas, it would be essential to identify gRNA(s) with a complementary sequence on the DNA (chromosome or plasmid) of the targeted strain only. The work by recently published Reuter and co-workers (Reuter et al., 2021) precisely reports a CSTB algorithm that allows identifying strain-/gene-specific gRNAs. These authors should discuss this point.

In my view, the most surprising point of the manuscript is (lines 171-175): "No resistance to the CRISPR module was detected by probing for bacteria harboring the combined resistance markers found on TP114 and in EcN KN02, indicating that all target cells that received eB-TP114::Kill1 were eliminated. The remaining <0.1% of target bacteria survived but did not acquire TP114::Kill1 based on their antibiotic resistance profile, raising the possibility that they could have been recalcitrant to conjugative transfer.". They go on with performing in vitro conjugation using the GFP reporter on the plasmid carrying the targeted cat gene. If I understand well, they conclude that all recipient cells surviving (0.1%) have not been reached by the killer plasmid (while all targeted recipient cells that receive the plasmid are killed). This is surprising since CRISPR-mediated killing systems usually give rise to a particular frequency of escaper mutants, i.e., recipients that evolve 'escaper' mutations allowing them to survive despite the acquisition of the killer plasmid. Escaper mutations have been shown to occur on the killer plasmid (inactivating the Cas9 or the gRNA), or on the targeted sequence (cat gene), thus avoiding recognition by the gRNA, either in the donor cell before transfer or in the recipient after transfer.
It would be a wonderful achievement to demonstrate unambiguously that the eB-TP114 prevents such escaper mutants' emergence. Because it is a critical point of the work, I would require one additional experiment to confirm that the eB-COP system avoids the emergence of escapers. What if the authors perform conjugation between the eB-COP strain (containing an eB-TP114 plasmid carrying a selectable resistance gene) and the targeted EcN KN03 strain in optimal conjugation conditions in vitro. The conjugation mix should be plated on a medium containing the antibiotic which resistance is carried by the eB-TP114 and chloramphenicol. This would most probably allow the isolation of viable CmR transconjugant cells (recipients having received the eB-TP114 but that can survive in the presence of Cm due to the acquisition of escaper mutations), even if their frequency of emergence is very low. Characterization of these escapers would require analyzing the sequence of the eB-TP114 and the cat-plasmid to identify escaper mutations, if any.

In any case, the authors should be commended for a nice and clean piece of work that undoubtedly contributes to the development of antibacterial strategies using conjugation-CRISPR plasmids.

Reviewer #3:

Summary
Neil et al. describes the engineering of a conjugative plasmid with high transfer rates in vitro and in vivo to deliver a CRISPR-Cas9 system into targeted bacteria. Using the TP114 plasmid, which they had previously discovered to have high rates of in vivo transduction, they use an accelerated evolution approach to improve the conjugative efficiency of this plasmid. Ultimately, to demonstrate the application of this approach they show efficacy in killing Citrobacter rodentium using a mouse model of infection.

General Remarks
This is a well written manuscript that achieves significant antibacterial effect in vivo using a murine model of C. rodentium infection, which I believe will be of interest to a general audience. As acknowledged by the authors, there have already been a number of reports using of CRISPR-Cas to target undesirable genes in bacteria as an antibacterial approach, using various mechanisms for delivery including phages (Citorik et al. Nat. Biotechnol. 2014; Bikard et al. Nat Biotechnol. 2014; Yosef et al. PNAS 2015) and plasmids (Hamilton et al. Nat Commun. 2019; Reuter et al. Nucleic Acids Res. 2021). Other studies have shown the use of CRISPR-Cas in vivo in the mouse gut either using a conjugative plasmid to deplete antibiotic resistance genes (Rodrigues et al. Antimicrob Agents Chemother. 2019) or a phage to deliver dCas to repress marker genes (Hsu et al. Nat Commun. 2020). In light of the previous work, it is the demonstration of C. rodentium elimination in the mouse gut that is the most substantive advance. However, my enthusiasm is tempered due to the absence of certain data and methods, which is described in more detail below.

Major Points
- In the section beginning in Line 82, it is indicated that the COP is specific for killing the targeted KN02 but not the non-targeted KN03 strain. I presume this is due to the gRNA-guided nuclease activity for the cat gene, but what is the efficiency of transconjugation? Do you see that both KN02 and KN03 are conjugated in vivo at the same frequency? Of those KN02 strains that remain and are not killed, do they survive because they did not receive the conjugative plasmid (but remain a possible recipient), developed resistance to conjugation, or developed resistance to the CRISPR/Cas nuclease (e.g., mutated cat gene)?

- Related to the above point, but more generally: other studies have shown that in situ conjugative transfer can be broadly targeting of diverse taxa, i.e., non-specific, in the gut (Ronda et al. Nat Methods. 2019). With a TP114 plasmid that has exceptionally high transfer rates in vivo, is it similarly broad and with high transfer rates for other species present in the background flora?

- In Line 99 and Extended Data Figure 3, this is the analysis of a streptomycin-treated gut microbiota and not an unperturbed microbiota. The effect of the antibiotic should be acknowledged. I believe the point of streptomycin treatment was to reduce colonization resistance so that the administered human commensal EcN strains (K01, K02, and K03) are able to stably engraft into the mouse gut. While this does not substantially affect anaerobes, it has substantial impact on facultative anaerobes. In this case, then wouldn't this also minimize interactions between the EcN strains and those that it would likely interact with in an unperturbed conventional microbiome (e.g., facultative anaerobes)? I would expect that the constant "suppression" of related species by streptomycin to mask any potential impact of the COP strain. To characterize the impact of the

COP strain under more realistic conditions, this analysis should be done using a murine commensal E. coli (i.e. without sustained antibiotic treatment). Alternatively, the related paragraph (especially Lines 108-110) should be modified to indicate that such analysis used a streptomycin-treated background.

- In vivo specificity assays (Figure 3), show an initial mix of target:non-target E. coli strains administered at a 1000:1 ratio. Considering a substantial bias towards the target strain and the requirement of physical interaction between donor and recipient for conjugation to occur, is it not more likely that the observed effect is simply due the odds with which the donor and recipient find each other in vivo? In other words, there seems like a 1000x greater chance that eB-COP would find the target KN02 rather than the non-target KN03, which seems to be a less-than ideal way to test the specificity of the approach, especially using the evolved TP114 plasmid with high transfer rates. Furthermore, while the targeted KN02 strain is depleted with a median lower than that of non-targeted KN03 strain, they do not appear to be significantly different and statistical analysis would be beneficial here.

- What are the fecal concentrations of eB-COP in Figures 3 and 4? Do they persist in the gut?

- Throughout the characterization of the efficacy of the COP strain, the gRNA targets an antibiotic resistance cassette that is genetically engineered into the bacterium. This is a reasonable approach for the characterization of the efficacy of the approach, but it is the same strategy in the final demonstration in vivo with C. rodentium, where the bacterium is engineered with a chloramphenicol cassette, which is also the target for the nuclease. How realistic would it be for an enteric pathogen to contain a chloramphenicol resistance cassette and as a more realistic demonstration in treating an infection, would it not be more realistic to target a core gene associated with C. rodentium?

- Related to an earlier comment, but it is especially important in the context of treatment of C. rodentium infection, what is the frequency of resistance this strategy?

- Lines 272-3 seem to imply that streptomycin was not used during the C. rodentium mouse model (Figure 4), but it was my understanding that streptomycin was used based on the figure caption. Then the materials and methods section indicates that streptomycin was only used in Figure 4c. Does this mean it was not used in the experiments for Figure 4d-f? If streptomycin was used in only certain cases of the mouse model and not all cases, why was it not applied throughout and what is the rationale? It would be helpful if the authors made the mouse models more clear by including experimental timelines for Figure 4 as well as when streptomycin was administered for Figures 1-3.

Minor Points

- In all box and whisker plots, the symbols for the individual data points are difficult to discern. Often, only two points can be identified, though there are certainly more present. Please make the symbols larger and more clearly visible.

- In line 240, "efficient" is used when I believe the authors intend to use "effective".

- In all figures that show concentrations of bacteria in stool (e.g., "CFU/mg feces") it would be helpful if it would also indicate the bacterium being quantified, such as "C. rodentium CFU/mg feces"

Response to reviewers

We would like to thank all reviewers for their thoughtful evaluation of our manuscript. The reviewers' comments are shown below in black while our responses are in blue. The format of the manuscript was also verified and modified to address the issues raised by the editor to follow the Molecular Systems Biology guidelines. We are confident that the modified version of the manuscript will now be appropriate for publication but remain open to any suggestion to improve our work.

Reviewer comments:

Reviewer #1:

Neil *et al.* report the engineering of a probiotic strain capable of transferring CRISPR-cas9 machinery to different bacterial species related to *E. coli* in the gastrointestinal tract. They achieve this by employing a conjugative plasmid previously identified by the authors in a different study and evolving this system to make it capable of high-efficiency DNA transfer. The authors then introduced this system into a probiotic *E. coli* strain. They used it to eliminate antibiotic-resistant *E. coli* and clear a *C. rodentium* infection, both in the mouse gut. This probiotic treatment approach does not seem to disturb the rest of the microbiota and has efficiencies comparable to conventional antibiotics in mice.

This study is an exciting demonstration of how molecular tools and systems biology methods can be employed to tackle the antibiotic resistance crisis. This study should be relevant to researchers in translational systems and synthetic biology. The conclusions are well supported by the experiments and the data presented.

We would like to thank Reviewer #1 for their nice summary of the manuscript, and we appreciate his/her careful review.

Major comments:

1) The authors do a great job explaining the way they engineer strain discrimination in simulated infection scenarios. However, they have not described how the CRISPR-cas9 kills the bacteria. The authors should elaborate on the precise killing mechanism triggered by the gene-editing machinery targeting antibiotic resistance genes. The manuscript focuses more on explaining that the gene transference occurs and that the target strain is eliminated. But there is no mention of the specific killing mechanism potentially caused by double-strand breaks in the target gene.

The killing mechanism used by CRISPR-Cas system has already been studied in other articles such as Cui and Bikard in 2016 (PMID: 27060147). A brief description of the CRISPR-Cas killing mechanism with the appropriate references is now included at lines 51-59 of the main manuscript.

2) How do the authors differentiate elimination of the antibiotic-resistant strain vs. elimination of specific genes? Because the assessment of the clearance is typically based on CFU counting in selective agar, is it possible that the CRISPR-Cas only caused a mutation in the selection gene but did not kill the bacteria?

The different selection mechanisms used to count CFU are also difficult to follow, making it challenging to find a clear answer to the above question.

This is an interesting question that was explored in Figure EV1b. In this experiment, TP114::Kill1 (targeting the *cat* gene conferring resistance to chloramphenicol) was transferred into *E. coli* KN02 (target strain containing the *cat* gene). Transconjugant CFUs were next selected and counted in the presence or absence of chloramphenicol in the medium. Similar numbers of *E. coli* KN02 transconjugants were obtained with or without chloramphenicol selection, indicating that cleavage of the *cat* gene by CRISPR-Cas9 resulted in cell death rather than loss of antibiotic resistance (which could have been observed if a mutation was present). This result was not originally presented in the main text but is now included at lines 109-114.

3) If the antibiotic-resistance gene is located on a plasmid, will the CRISPR-Cas-based killing strategy still work? Are plasmid- or chromosome-based resistance genes more common in clinical setting? The authors should discuss any limitation of the CRISPR-Cas strategy to target natural pathogenic bacteria.

When located on a plasmid, CRISPR-Cas9 cleavage will most likely result in the loss of the plasmid, as evidenced in the NO-SCAR genome engineering method (Reisch and Prater 2015, PMID: 26463009) and in Dong *et al*. 2019 (PMID: 30267758) (see line 57-59). This can in turn lead to the death of their host if toxin/anti-toxin systems or other mechanisms are present to ensure plasmid maintenance. However, in some more specific cases where the targeted resistance gene is located between repeated DNA sequences such as in integrons, CRISPR-Cas9 targeting would potentially result in the loss of antibiotic resistance through homologous-recombination-based double-strand break repair (Roy *et al*., 2020; PMID: 32556263).

Antibiotic resistance genes can be found in the chromosome or plasmids depending on the strains of interest. A precise quantification of plasmid vs chromosome encoded antibiotic resistance in clinical settings is a complex endeavour that will depend on many factors. We have not found a clear answer for this question in the literature.

There are a few potential limitations of the CRISPR-Cas strategy to target natural pathogenic bacteria. First, the pathogenic bacteria must be recognized by the conjugative machinery and the transfer rates must be sufficiently high to have a significant impact on the targeted population (mentioned at lines 59-61 and 71-79 of the manuscript). Potential plasmid surface or entry exclusion mechanisms, rapid degradation of transferred DNA by restriction enzymes or other systems could impact the delivery of the CRISPR-*cas9* system. Intracellular pathogen could also be more difficult to reach for the COP bacterium. Second, the CRISPR-Cas9 system has to be efficiently expressed and active in the target bacterium. The promoter driving the expression of the CRISPR-Cas9 genes must allow expression in the target bacterium. The presence of appropriate CRISPR-Cas9 inhibitors encoded by the target bacterium, although not frequently observed in E*nterobacteriaceae* (Hwang and Maxwell, 2019 PMID: 31021234), could also lower the treatment efficiency. Third, the sequences targeted by the CRISPR-Cas9 system must be carefully selected to ensure that certain alleles could make a fraction of the population insensitive to the treatment.

These limitations and in some cases potential strategies that could be used to enable efficient elimination of natural pathogens are now discussed in the main manuscript (see lines 278-284, 289-314, and 352-356).

4) Line 107-110. The language lacks precision. For instance, “remained similar between the controls and the COP” is unclear. The data is rather complex, and it is unclear how “similarity” is quantified or justified.

Can the authors support their statement using quantitative metrics? The analysis is critical because the authors repeatedly claim that their method "leaves the rest of the microbiota essentially untouched".

This section has been rewritten to improve clarity with a more precise language and to report quantitative metrics directly in the text (lines 145-148). We did not observe any global effect on the microbiota using conventional metrics or approaches (alpha and beta variances, PCA, PCoA).

Minor comments:

5) The authors should discuss possible clearance strategies for the COP strains after the infection has been eliminated. In biosafety consideration, isn't a genetically modified bacterial species typically disabled from conjugation to prevent any horizontal gene transfer? Is the authors' strategy consistent with the current safety regulation? If not, the authors should discuss future challenges of their strategy in term of biosafety and clinical application.

Our work was focused on maximizing transfer rates to test if the COP strategy had the potential to clear an infection. The COP strain, as presented in this manuscript, is not yet ready for clinical applications but critical biocontainment measures will certainly have to be considered to limit the COP donor strain survival and the persistence of the transferred DNA molecule in the environment. We now discuss biocontainment strategies at lines 346-352.

6) The labeling of the Nissle strains (KN01, KN02, KN03, EcN KN01 + TP114, etc.) is confusing. The authors might want to rename these strains with more informative names to prevent the reader from going back and forth between the main text and the supplementary material to find the information on the genotype and phenotype. It will also be great to include more descriptive information in the figure labels.

Unfortunately, changing the strain names will be challenging at this point as they were already published under these names in our previous work (Neil *et al*. 2020, PMID 32963323). However, we now include a brief description of each strain at lines 93-96 to increase readability, and moved the Appendix Table S1 into the main manuscript (now Table EV1) to allow easier access to strain information. We also revised all figure labels to ensure that all essential information and details are given to the readers.

7) Why is there a difference in the target/non-target ratio between Figs 1 and 3 (1:1 and 1000:1, respectively)? If the authors are trying to demonstrate that the eB-COP strain performs better than the COP strain, shouldn't the ratios be kept the same?

We understand that preserving a ratio of 1:1 would in principle facilitate the comparison between the COP and eB-COP strains (shown respectively in Figure 1 and Figure 3). We nonetheless opted to change ratios between the target and non-target strain from 1:1 to 1000:1 for this assessment of the eB-COP. The main reason was that, if the eB-COP system had a drastically improved activity, the 1:1 ratio could limit the experimentally observable dynamic range of target strain elimination compared to the 1000:1 ratio. In other words, the elimination of the target strain could have been limited by its abundance rather than by the activity of the eB-COP compared to the COP. We also sought to take advantage of this experiment to observe how the elimination of the target strain would impact the non-target strain population since a larger proportion of its niche would become available. Finally, we would like to highlight the fact that Figure 4 presents a direct comparison of the COP and eB-COP systems in the *Citrobacter rodentium* infection model, and that only the eB-COP treatment resulted in full clearance of *Citrobacter*.

Reviewer #2:

I review this article non-anonymously, Christian Lesterlin Cell-to-Cell DNA transfer lab (CNRS, Molecular microbiology and Structural biochemistry).

In the manuscript "High-efficiency Delivery of CRISPR-Cas9 by engineered probiotics enables precise microbiome editing", Neil and co-workers investigate the possibility of using conjugation plasmids to deliver CRISPR systems that exert antibacterial activity. This promising strategy has been the focus of several recent publications in the past few years to target E. coli (Citorik et al., 2014; Dong et al., 2019; Ruotsalainen et al., 2019; Wang et al., 2019), Salmonella Typhimurium (Hamilton et al., 2019) or Enterococcus faecalis (Rodrigues et al., 2019). The authors should also cite and discuss the recent article by Reuter and co-workers (Reuter et al., 2021), which probably came out after the finalization of the present manuscript.

We added the citation for the publication by Reuter *et al.* 2021, which was indeed published after the finalization of our manuscript.

There are several bottlenecks to overcome to validate this type of approach as a practical non-antibiotic antibacterial approach for in situ microbiota manipulation/editing. One major limitation is the efficiency of plasmids transfer in situ. In this work, the authors specifically tackle this question by performing Accelerated Laboratory Evolution (ALE), resulting in the successful isolation of TP114 mutants that exhibit enhanced transfer rates. In the gut, the eB-TP114 clone (eB527) exhibits improved transfer compared to the native TP114. The conjugation system of the eB-TP114 has then been chosen to enhance the efficiency of the COP system in further mouse gut experiments. Remarkably, the authors convincedly show that the eB-COP plasmid allows the elimination of drug-resistant *Escherichia coli* and the mouse pathogen *Citrobacter rodentium* in the mouse gut.

The manuscript is concise and well written, the experiments are performed to a high standard, and the results are well presented in the figures. This is a very nice manuscript and the presented conclusions follow from careful analysis of the data. As it is, the manuscript is of appropriate quality and significance for publication. Nonetheless, I propose to consider some minor changes and I suggest/recommend only one additional experiment, which would be required to ascertain the observation regarding the absence of escaper mutants (see last point below).

We thank Dr. Christian Lesterlin for his outstanding transparency and insightful review.

Specific points:

1) I think it would be very helpful to add the genetic map of the killer plasmid to the main figure 1, for the reader to grasp the COP system in one glance.

We carefully considered this suggestion, but we believe that the genetic map of the COP plasmid comprising the CRISPR-Cas9 system would be very small and difficult to read in Figure 1. Also, the construction of the COP strain was previously described in Neil *et al.* 2019, where we described the DROID technique used to load CRISPR on TP114 in many details.

2) Identification of TP114 "superspreadrer" variants is a most significant achievement. It provides a

conjugation system that performs high transfer efficiency in the mouse gut, at least between E. coli donors and E. coli/C rodentium recipients. TP114 variants carry mutations in the TP114-084/TP114-085 intergenic region. The eB-TP114 mutants were additionally enriched with mutations in a predicted transcription regulator yaeC, whereas "eA-TP114 mutants displayed an increased number of deleterious mutations in the pil genes compared to eB-TP114". In the gut, the eB-TP114 clone (eB527) (but not the eA-TP114 clone (eA517)) exhibits improved transfer compared to TP114.
I agree that, as stated by the authors (line 259): "The implication of these genes for TP114 conjugation will require more investigation". Nonetheless, it would be interesting for the reader to have more details regarding the author's interpretation of the predicted/hypothetical impact of these mutations of the conjugation rates (possible role of yaeC mutations and other mutations). The authors should then consider elaborating a bit more on this point in the discussion.

We have added some details in the discussion (see lines 321-329) about the impact of the mutations found in the eB-COP. We are currently investigating this aspect of the eB-COP system and plan to publish these results in the coming year.

3) In the same idea, I think that the statement (line 275): "The eB-COP with its constitutively active transfer machinery would have a more decisive impact on the infection." should be rephrases. It is likely indeed that the expression of the conjugation machinery is affected (upregulated), yet at this stage, this is not the only possible explanation for the observed enhance transfer of the mutated TP114.

We agree that at this stage this sentence should be reworded to ensure that other explanations are not excluded and have made changes accordingly (see line 341-342).

4) Since several antibacterial systems based on conjugation and CRISPR have been published, the authors should discuss and emphasize their methodology's pros and cons compared to previous reports. In particular, they should explain the choice of using autonomous conjugative plasmids instead of smaller mobilizable plasmids, which can be transferred using the conjugation machinery encoded in trans on an additional helper plasmid on the chromosome of the donor strain. It could also be appreciated to have a few words on the biocontainment aspects regarding the uncontrolled propagation of such antibacterial plasmids that could be used in therapeutic treatments.

The main focus of our manuscript was to obtain a DNA delivery system sufficiently efficient to allow clearance of target bacteria from the gut microbiota. We thus opted for a system configuration that we hypothesized to be the most efficient (*cis* mobilization of the CRISPR-*cas9* system). However, other configurations are possible (*in trans* mobilization of shuttle plasmids harboring the CRISPR-*cas9* system, for example) but this strategy requires the identification of the TP114 origin of transfer, which was not available during these experiments. This information is now mentioned at lines 349-352 of the manuscript. We now also briefly discuss of biocontainment in the discussion (lines 346-350).

5) While developing tools for microbiome editing, it is critically important to ensure that the CRISPR systems will be active in the target strain only and have no impact on the other species present in the microbiota). To that end, the authors use metagenomic analysis of the microbiota composition and show that COP had no significant effect on the microbiota diversity. Yet, in the experiments presented, mice were initially treated with Streptomycin (1g/L), which results in the "simplification" of the composition of their microbiota in terms of microbial diversity. To achieve strain-specific targeting in more complex natural microbiotas, it would be essential to identify gRNA(s) with a complementary sequence on the DNA (chromosome or plasmid) of the targeted strain only. The work by recently published Reuter and co-

workers (Reuter et al., 2021) precisely reports a CSTB algorithm that allows identifying strain-/gene-specific gRNAs. These authors should discuss this point.

This is an excellent point that is now discussed in the manuscript at lines 285-303.

6) In my view, the most surprising point of the manuscript is (lines 171-175): "No resistance to the CRISPR module was detected by probing for bacteria harboring the combined resistance markers found on TP114 and in EcN KN02, indicating that all target cells that received eB-TP114::Kill1 were eliminated. The remaining <0.1% of target bacteria survived but did not acquire TP114::Kill1 based on their antibiotic resistance profile, raising the possibility that they could have been recalcitrant to conjugative transfer.". They go on with performing in vitro conjugation using the GFP reporter on the plasmid carrying the targeted cat gene. If I understand well, they conclude that all recipient cells surviving (0.1%) have not been reached by the killer plasmid (while all targeted recipient cells that receive the plasmid are killed). This is surprising since CRISPR-mediated killing systems usually give rise to a particular frequency of escaper mutants, i.e., recipients that evolve 'escaper' mutations allowing them to survive despite the acquisition of the killer plasmid. Escaper mutations have been shown to occur on the killer plasmid (inactivating the Cas9 or the gRNA), or on the targeted sequence (cat gene), thus avoiding recognition by the gRNA, either in the donor cell before transfer or in the recipient after transfer.
It would be a wonderful achievement to demonstrate unambiguously that the eB-TP114 prevents such escaper mutants' emergence. Because it is a critical point of the work, I would require one additional experiment to confirm that the eB-COP system avoids the emergence of escapers. What if the authors perform conjugation between the eB-COP strain (containing an eB-TP114 plasmid carrying a selectable resistance gene) and the targeted EcN KN03 strain in optimal conjugation conditions in vitro. The conjugation mix should be plated on a medium containing the antibiotic which resistance is carried by the eB-TP114 and chloramphenicol. This would most probably allow the isolation of viable CmR transconjugant cells (recipients having received the eB-TP114 but that can survive in the presence of Cm due to the acquisition of escaper mutations), even if their frequency of emergence is very low. Characterization of these escapers would require analyzing the sequence of the eB-TP114 and the cat-plasmid to identify escaper mutations, if any.

The presence of escapers and the mechanism of resistance represent an interesting topic that we have begun to explore. No escapers were detected in the experiment described at lines 210-212 (Figure 3). However, COP escaper mutants were occasionally observed *in vitro* and *in situ*. These escapers were sequenced and all contained mutated copies of either the *cas9* gene or the gRNA. We did not identify any mutation in the *cat* gene in these experiments. This suggests that COP system escapers acquired a mutated copy of the CRISPR-*cas9* system and were next protected from receiving a functional copy of the *CRISPR-cas9* system by conjugative transfer exclusion mechanisms. These data are now presented in a new additional Appendix Figure S1 and discussed in the manuscript at lines 131-136 A paragraph was also added in the Materials and Methods section to describe the analysis of COP escapers mutants (see lines 443-458).

Interestingly, a low frequency of eB-COP escapers mutants was also observed *in vitro* (similar to the escaper rate observed with the COP system) but never *in situ* (either with *E. coli* or *C. rodentium*). We are still actively investigating this observation, but it is possible that the eB-COP could transfer more than one copy of itself per conjugation event, hence lowering the chance of receiving only a mutated copy of the CRISPR system. Indeed, TP114 was found to be maintained at 1-7 copies per cell *in vitro* (unpublished results), and the donor strain could potentially harbor a mix of inactivated and functional copies of the CRISPR module in the same bacterium. There is also the possibility that plasmid exclusion could be more

frequently bypassed by the eB-COP, allowing repeated transfer in the target cell from different donor cells, and thus decreasing the chance of receiving an inactive CRISPR-*cas9* system. In any case, additional work will be needed to better characterize the mechanisms that influence the apparition of escapers.

7) In any case, the authors should be commended for a nice and clean piece of work that undoubtedly contributes to the development of antibacterial strategies using conjugation-CRISPR plasmids.

We thank Pr. Lesterlin for his thoughtful review of our manuscript.

Reviewer #3:

Summary
Neil et al. describes the engineering of a conjugative plasmid with high transfer rates in vitro and in vivo to deliver a CRISPR-Cas9 system into targeted bacteria. Using the TP114 plasmid, which they had previously discovered to have high rates of in vivo transduction, they use an accelerated evolution approach to improve the conjugative efficiency of this plasmid. Ultimately, to demonstrate the application of this approach they show efficacy in killing Citrobacter rodentium using a mouse model of infection.

We would like to first thank Reviewer #3 for her/his careful review of our manuscript.

General Remarks

This is a well written manuscript that achieves significant antibacterial effect in vivo using a murine model of C. rodentium infection, which I believe will be of interest to a general audience. As acknowledged by the authors, there have already been a number of reports using of CRISPR-Cas to target undesirable genes in bacteria as an antibacterial approach, using various mechanisms for delivery including phages (Citorik et al. Nat. Biotechnol. 2014; Bikard et al. Nat Biotechnol. 2014; Yosef et al. PNAS 2015) and plasmids (Hamilton et al. Nat Commun. 2019; Reuter et al. Nucleic Acids Res. 2021). Other studies have shown the use of CRISPR-Cas in vivo in the mouse gut either using a conjugative plasmid to deplete antibiotic resistance genes (Rodrigues et al. Antimicrob Agents Chemother. 2019) or a phage to deliver dCas to repress marker genes (Hsu et al. Nat Commun. 2020). In light of the previous work, it is the demonstration of C. rodentium elimination in the mouse gut that is the most substantive advance. However, my enthusiasm is tempered due to the absence of certain data and methods, which is described in more detail below.

Major Points:

1) In the section beginning in Line 82, it is indicated that the COP is specific for killing the targeted KN02 but not the non-targeted KN03 strain. I presume this is due to the gRNA-guided nuclease activity for the cat gene, but what is the efficiency of transconjugation? Do you see that both KN02 and KN03 are conjugated in vivo at the same frequency? Of those KN02 strains that remain and are not killed, do they survive because they did not receive the conjugative plasmid (but remain a possible recipient), developed resistance to conjugation, or developed resistance to the CRISPR/Cas nuclease (e.g., mutated cat gene)?

EcN KN02 and EcN KN03 are highly similar strains differing only by their selective markers. Both strains display virtually identical TP114 transconjugant formation rates *in vitro* and *in situ*. However, when the

CRISPR module is transferred, the rate of conjugation into KN02 drops to or near to zero because of EcN KN02 target cell death (Figure EV1). As mentioned in the response to Reviewer #2, we now include additional details on the resistance phenomenon. As discussed above, the COP treatment has a low level of resistance with escapers that arise *in vitro* as well as in certain mice (Appendix Figure S1). This is now detailed in lines 109-114 and 131-136.

2) Related to the above point, but more generally: other studies have shown that in situ conjugative transfer can be broadly targeting of diverse taxa, i.e., non-specific, in the gut (Ronda et al. Nat Methods. 2019). With a TP114 plasmid that has exceptionally high transfer rates in vivo, is it similarly broad and with high transfer rates for other species present in the background flora?

The work done by Ronda *et al.* used RP4, a plasmid known for its broad host range that in our hands did not transfer at high efficiency *in situ*. Unfortunately, Ronda *et al*. did not report conjugation rates in a transconjugants/recipient or transconjugants/donor format in their study, so it is difficult to know the rate at which their construct disseminates in other bacteria. In the case of TP114, $IncI_2$ plasmids are generally thought to have a relatively limited host-range (Suzuki *et al*., 2010; PMID: 20851899). We are currently performing experiments to characterize the host range of TP114 and will report the data in a separate manuscript when available.

3) In Line 99 and Extended Data Figure 3, this is the analysis of a streptomycin-treated gut microbiota and not an unperturbed microbiota. The effect of the antibiotic should be acknowledged. I believe the point of streptomycin treatment was to reduce colonization resistance so that the administered human commensal EcN strains (K01, K02, and K03) are able to stably engraft into the mouse gut. While this does not substantially affect anaerobes, it has substantial impact on facultative anaerobes. In this case, then wouldn't this also minimize interactions between the EcN strains and those that it would likely interact with in an unperturbed conventional microbiome (e.g., facultative anaerobes)? I would expect that the constant "suppression" of related species by streptomycin to mask any potential impact of the COP strain. To characterize the impact of the COP strain under more realistic conditions, this analysis should be done using a murine commensal E. coli (i.e. without sustained antibiotic treatment). Alternatively, the related paragraph (especially Lines 108-110) should be modified to indicate that such analysis used a streptomycin-treated background.

Streptomycin treatment was indeed needed in our experiment to facilitate the colonization of EcN derived strains for experiments presented in Figure 1 and Figure 3. Please note that the COP and eB-COP treatment in the *C. rodentium* model did not include streptomycin in the mouse drinking water. We have carefully modified the text to clearly indicate when mice were treated with streptomycin. Changing the donor strain for a murine *E. coli* isolate is a very good suggestion that we plan to investigate in future work.

4) In vivo specificity assays (Figure 3), show an initial mix of target:non-target E. coli strains administered at a 1000:1 ratio. Considering a substantial bias towards the target strain and the requirement of physical interaction between donor and recipient for conjugation to occur, is it not more likely that the observed effect is simply due the odds with which the donor and recipient find each other in vivo? In other words, there seems like a 1000x greater chance that eB-COP would find the target KN02 rather than the non-target KN03, which seems to be a less-than ideal way to test the specificity of the approach, especially using the evolved TP114 plasmid with high transfer rates. Furthermore, while the targeted KN02 strain is

depleted with a median lower than that of non-targeted KN03 strain, they do not appear to be significantly different and statistical analysis would be beneficial here.

The reviewer is right that the chance of a donor bacterium interacting with a target or non-target bacterium will be affected by their ratio, but this phenomenon is more complex to model than a simple linear relationship (Krone *et al.* 2007; PMID: 17660444, Zhong *et al.* 2012; PMID: 22085738, Volkova *et al.* 2013; PMID: 23982723). It is therefore difficult to provide statistical analyses to respond to this comment. As mentioned in the response to point #7 of Reviewer #1, the main reason for this was that the 1:1 ratio could limit the experimentally observable dynamic range of target strain elimination compared to the 1000:1 ratio. In addition, a direct comparison of the COP and eB-COP was performed in the *C. rodentium* infection model and clearly demonstrated the superiority of the eB-COP (Figure 4).

5) What are the fecal concentrations of eB-COP in Figures 3 and 4? Do they persist in the gut?

For Figure 3, the eB-COP concentration in feces was similar to the target strain on day 1 post-treatment but quickly dropped near the detection limit starting day 2 post treatment. This can be attributed to the high density of competing strain (target and non-target strains) already present when the eB-COP strain was introduced. The eB-COP strain was found to be colonizing at a lesser extent than the COP strain, possibly due to a higher metabolic burden of the eB-TP114 plasmid. The continued effect of eB-COP treatment in Figure 3 was likely due to the presence of non-target strain transconjugants disseminating the eB-COP construct.

For Figure 4, we took into account the low colonization level of the eB-COP in our administration routine. Since the eB-COP was administered daily throughout the experiment, high levels of eB-COP were found at all timepoints. However, it is expected that in an undisturbed microbiota, the eB-COP strain will not be able to colonize for extended periods of time. This is now briefly discussed at lines 256-258.

6) Throughout the characterization of the efficacy of the COP strain, the gRNA targets an antibiotic resistance cassette that is genetically engineered into the bacterium. This is a reasonable approach for the characterization of the efficacy of the approach, but it is the same strategy in the final demonstration in vivo with C. rodentium, where the bacterium is engineered with a chloramphenicol cassette, which is also the target for the nuclease. How realistic would it be for an enteric pathogen to contain a chloramphenicol resistance cassette and as a more realistic demonstration in treating an infection, would it not be more realistic to target a core gene associated with C. rodentium?

We have chosen to keep the gRNA targeting the *cat* gene in the *C. rodentium* infection model to isolate the DNA transfer rate as the main variable throughout our experiments. Previous work has shown that different gRNA can have strikingly different efficiencies. Changing the gRNA for the experiment presented in Figure 4 could have introduced a confounding effect. Future development of microbiome editing or anti-bacterial technologies using CRISPR-Cas9 delivery by bacterial conjugation will have to target conserved genes directly in the chromosome of the targeted strains but we considered that this was beyond the scope of the current manuscript. This aspect was discussed in more details in the response to point #3 of Reviewer #1.

7) Related to an earlier comment, but it is especially important in the context of treatment of C. rodentium infection, what is the frequency of resistance this strategy?

The frequency of resistance and the most likely mechanisms are now described in Appendix Figure S1. While escapers were detected with the COP system *in vivo* (see Appendix Figure S1 and response to point #6 raised by Reviewer #2), no escapers were isolated during *in situ* experiments using the eB-COP strain.

8) Lines 272-3 seem to imply that streptomycin was not used during the C. rodentium mouse model (Figure 4), but it was my understanding that streptomycin was used based on the figure caption. Then the materials and methods section indicates that streptomycin was only used in Figure 4c. Does this mean it was not used in the experiments for Figure 4d-f? If streptomycin was used in only certain cases of the mouse model and not all cases, why was it not applied throughout and what is the rationale? It would be helpful if the authors made the mouse models more clear by including experimental timelines for Figure 4 as well as when streptomycin was administered for Figures 1-3.

We have adjusted the main text, Figure captions, and Materials and Methods sections to clearly indicate when streptomycin was used (see lines 139, 193, 240-241, 247-248, 713, 742 and 765-766). Briefly, streptomycin treatment for Figure 1-3 was added to mice drinking water throughout these experiments. For the *C. rodentium* infection model (Figure 4), mice were not treated with streptomycin except for panel 4c that provides a comparison between the COP, eB-COP and a streptomycin treatment. No streptomycin was added to mice drinking water in Figure 4a, 4b, 4d, 4e, and 4f. The reason for using streptomycin in experiments reported in Figures 1-3 was to facilitate the establishment of EcN derivatives that are not particularly well equipped to colonize the mouse intestinal microbiota. However, *C. rodentium* robustly infects mice and did not require any streptomycin treatment for proper colonization. This information is now provided at lines 240-241 and 247-248 of the manuscript.

<u>Minor Points</u>

9) In all box and whisker plots, the symbols for the individual data points are difficult to discern. Often, only two points can be identified, though there are certainly more present. Please make the symbols larger and more clearly visible.

The datapoints were enlarged in all box and whiskers plot.

10) In line 240, "efficient" is used when I believe the authors intend to use "effective".

The text was modified accordingly.

11) In all figures that show concentrations of bacteria in stool (e.g., "CFU/mg feces") it would be helpful if it would also indicate the bacterium being quantified, such as "C. rodentium CFU/mg feces"

We have made the modifications as requested.

Thank you for sending us your revised manuscript. We have now heard back from the two reviewers who agreed to evaluate your study. As you will see, the reviewers are overall satisfied with the modifications made and think that the study is now suitable for publication.

Before we can formally accept your manuscript, we would ask you to address the following issues:

1. please address the remaining minor issues raised by Reviewer #3 as well as his/her comment about providing the genetic map of COP and the availability of the plasmid and its sequence.

On a more editorial level.

REFEREE REPORTS

Reviewer #1:

The authors have addressed all my comments.

Reviewer #3:

The authors have nicely addressed my concerns and I believe the manuscript is ready for publication. However, I do ask for a couple of points of clarification:

>In lines 213-214, the authors revise their text to indicate that "No resistance to the CRISPR module was detected". Can they also indicate the level of sensitivity in their assay?

>In the materials and methods, could the authors indicate the conditions used to differentiate

between the different strains cultured onto MacConkey plates? In their response to my earlier question (Referee 3, Question 5) they indicated the presence of consistently high levels of eB-COP in stool during C rodentium colonization (Figure 4E-4F), but with the description in the materials and methods it is unclear how they quantified only C. rodentium on MacConkey plates without also counting eB-COP (both would grow).

As we did not manage to get Reviewer #2's input on the revisions, we asked Reviewer #3 to look at your responses to Reviewer #2's comments. I include below the feedback from Reviewer #3.

Reviewer #3

I think the authors largely satisfied Reviewer #2's comments, except in regards to Specific point #1. Reviewer #2 asks for a genetic map of the COP plasmid but the authors decline to include it in Figure 1. I understand the authors' rationale for not including it into Figure 1, but I also agree with Reviewer #2 that a genetic map would be very helpful. The authors should include genetic maps of the plasmids somewhere, maybe the supplementary figures, since they play a prominent role in the manuscript.

Also, I see in the Data Availability section that the sequences of the plasmids have not been made available. I do not know if this is in compliance with MSB policy, but I would think that the plasmid and its sequence should be deposited into repositories.

Thank you for sending us your revised manuscript. We have now heard back from the two reviewers who agreed to evaluate your study. As you will see, the reviewers are overall satisfied with the modifications made and think that the study is now suitable for publication.

Before we can formally accept your manuscript, we would ask you to address the following issues:

Thank you for your careful revision of our manuscript. Please find below our response to the issues raised by both the editorial team and the reviewers. We are confident that we have adequately addressed all points, which further improved our manuscript.

Reviewer #1:

The authors have addressed all my comments.

We are thankful to reviewer 1 for their time and considerations.

Reviewer #3:

The authors have nicely addressed my concerns and I believe the manuscript is ready for publication. However, I do ask for a couple of points of clarification:

We would like to thank reviewer 3 who not only took the time to review our modifications in response to his/her comments but also accepted to revise the concerns raised by reviewer 2.

>In lines 213-214, the authors revise their text to indicate that "No resistance to the CRISPR module was detected". Can they also indicate the level of sensitivity in their assay?

The detection limit for this test varies between mice and through time as it is dependent on the colonization levels of the target bacteria. We added in the main text the approximate detection limit (~10^-6) for all feces analyzed.

>In the materials and methods, could the authors indicate the conditions used to differentiate between the different strains cultured onto MacConkey plates? In their response to my earlier question (Referee 3, Question 5) they indicated the presence of consistently high levels of eB-COP in stool during C rodentium colonization (Figure 4E-4F), but with the description in the materials and methods it is unclear how they quantified only C. rodentium on MacConkey plates without also counting eB-COP (both would grow).

We clarified this point in the methods section (see lines 535-537). Reviewer 3 is correct that both *E. coli* and *C. rodentium* grow on MacConkey plates, although with a slightly different morphology. We used a streptomycin and chloramphenicol resistant variant of *C. rodentium* in our experiments while the *E. coli* donor strain was resistant to streptomycin and spectinomycin. We used a combination of streptomycin + chloramphenicol plates to select *C. rodentium* (chloramphenicol inhibits the growth of the donorstrain) and spectinomycin + kanamycin plate to select our donor strain (both of which inhibits the growth of our target *C. rodentium* strain. Transconjugants were selected on plates containing streptomycin, chloramphenicol and kanamycin as kanamycin resistance is mobilized by TP114.

I think the authors largely satisfied Reviewer #2's comments, except in regards to Specific point #1. Reviewer #2 asks for a genetic map of the COP plasmid but the authors decline to include it in Figure 1. I understand the authors' rationale for not including it into Figure 1, but I also agree with Reviewer #2 that a genetic map would be very helpful. The authors should include genetic maps of the plasmids somewhere, maybe the supplementary figures, since they play a prominent role in the manuscript.

Also, I see in the Data Availability section that the sequences of the plasmids have not been made available. I do not know if this is in compliance with MSB policy, but I would think that the plasmid and its sequence should be deposited into repositories.

The plasmids were previously deposited on Genbank as part of another publication from our group. The accession numbers were shown in the referenced article. To improve access to the information, we added those accession numbers in Table EV1. We also included a graphic map of the pKill1 plasmid and of its insertion in TP114 in the new Appendix Figure S1. References to the other Appendix figures were adjusted in the text accordingly. In relation to plasmid availability, TP114 is publicly available through DSMZ. Other plasmids are available upon request for the moment and will be made available at Addgene.

Accepted **13th Sep 2021**

Thank you again for sending us your revised manuscript. We are now satisfied with the modifications made and I am pleased to inform you that your paper has been accepted for publication.

EMBO PRESS

YOU MUST COMPLETE ALL CELLS WITH A PINK BACKGROUND 🡇

PLEASE NOTE THAT THIS CHECKLIST WILL BE PUBLISHED ALONGSIDE YOUR PAPER

Corresponding Author Name: Sébastien Rodrigue
Journal Submitted to: Molecular Systems Biology
Manuscript Number: MSB-2021-10335

USEFUL LINKS FOR COMPLETING THIS FORM

http://www.antibodypedia.com
http://1degreebio.org
http://www.equator-network.org/reporting-guidelines/improving-bioscience-research-repor
http://grants.nih.gov/grants/olaw/olaw.htm
http://www.mrc.ac.uk/Ourresearch/Ethicsresearchguidance/Useofanimals/index.htm
http://ClinicalTrials.gov
http://www.consort-statement.org
http://www.consort-statement.org/checklists/view/32-consort/66-title
http://www.equator-network.org/reporting-guidelines/reporting-recommendations-for-tumo
http://datadryad.org
http://figshare.com
http://www.ncbi.nlm.nih.gov/gap
http://www.ebi.ac.uk/ega
http://biomodels.net/
http://biomodels.net/miriam/
http://jjj.biochem.sun.ac.za
https://osp.od.nih.gov/biosafety-biosecurity-and-emerging-biotechnology/
http://www.selectagents.gov/

Reporting Checklist For Life Sciences Articles (Rev. June 2017)

This checklist is used to ensure good reporting standards and to improve the reproducibility of published results. These guidelines are consistent with the Principles and Guidelines for Reporting Preclinical Research issued by the NIH in 2014. Please follow the journal's authorship guidelines in preparing your manuscript.

A- Figures

1. Data

The data shown in figures should satisfy the following conditions:

- the data were obtained and processed according to the field's best practice and are presented to reflect the results of the experiments in an accurate and unbiased manner.
- figure panels include only data points, measurements or observations that can be compared to each other in a scientifically meaningful way.
- graphs include clearly labeled error bars for independent experiments and sample sizes. Unless justified, error bars should not be shown for technical replicates.
- if n< 5, the individual data points from each experiment should be plotted and any statistical test employed should be justified
- Source Data should be included to report the data underlying graphs. Please follow the guidelines set out in the author ship guidelines on Data Presentation.

2. Captions

Each figure caption should contain the following information, for each panel where they are relevant:

- a specification of the experimental system investigated (eg cell line, species name).
- the assay(s) and method(s) used to carry out the reported observations and measurements
- an explicit mention of the biological and chemical entity(ies) that are being measured.
- an explicit mention of the biological and chemical entity(ies) that are altered/varied/perturbed in a controlled manner.
- the exact sample size (n) for each experimental group/condition, given as a number, not a range;
- a description of the sample collection allowing the reader to understand whether the samples represent technical or biological replicates (including how many animals, litters, cultures, etc.).
- a statement of how many times the experiment shown was independently replicated in the laboratory.
- definitions of statistical methods and measures:
  - common tests, such as t-test (please specify whether paired vs. unpaired), simple χ2 tests, Wilcoxon and Mann-Whitney tests, can be unambiguously identified by name only, but more complex techniques should be described in the methods section;
  - are tests one-sided or two-sided?
  - are there adjustments for multiple comparisons?
  - exact statistical test results, e.g., P values = x but not P values < x;
  - definition of 'center values' as median or average;
  - definition of error bars as s.d. or s.e.m.

Any descriptions too long for the figure legend should be included in the methods section and/or with the source data.

In the pink boxes below, please ensure that the answers to the following questions are reported in the manuscript itself. Every question should be answered. If the question is not relevant to your research, please write NA (non applicable). We encourage you to include a specific subsection in the methods section for statistics, reagents, animal models and human subjects.

B- Statistics and general methods

Please fill out these boxes 🡇 (Do not worry if you cannot see all your text once you press return)

1.a. How was the sample size chosen to ensure adequate power to detect a pre-specified effect size?	All experiments performed under laboratory conditions that did not involve animal samples were done in independent biological triplicates (Figures 2, 4a, EV1, EV3a, EV4). Biological triplicates were used in all conjugation experiments, including evolution experiments, to ensure reproductibility.
1.b. For animal studies, include a statement about sample size estimate even if no statistical methods were used.	All animal studies included at least four individual mice per test group to ensure sufficient statistical power for data analysis. Mice experiments were designed to give complementary information on the system and more individuals were added in mice groups when appropriate. Experiments presented in Figure 1 were done in two independent mice studies of 4 individuals.
2. Describe inclusion/exclusion criteria if samples or animals were excluded from the analysis. Were the criteria pre-established?	Mice were excluded from analysis when the recipient bacteria failed to colonize the GI tract as pre-established. Mice were also excluded from analysis when they had to be sacrificed due to health issues related to manipulations or observed prior to experiment in accordance with our faculty animal care commitee. No mice were excluded due to symptoms related to Citrobacter rodentium infection (Figure 4).
3. Were any steps taken to minimize the effects of subjective bias when allocating animals/samples to treatment (e.g. randomization procedure)? If yes, please describe.	Animals were randomly distributed into treatment groups by an unknowing personnel. Experiments involving the Citrobacter infection model were performed using a blind study design. The person in charge of following the symptoms had no knowledge of the treatments administered to the mice, nor did the person in charge of counting the CFU on Citrobacter plates.
For animal studies, include a statement about randomization even if no randomization was used.	Mice were distributed in cages upon arrival at the animal facility in a completely random manner by an animal technician that had no knowledge of the experiment.
4.a. Were any steps taken to minimize the effects of subjective bias during group allocation or/and when assessing results (e.g. blinding of the investigator)? If yes please describe.	A blind study design was applied for all Citrobacter experiments as well as for mice randomization upon arrival. Symptoms were evaluated by animal technician without prior knowledge on the treatment administered to mice. Treatments were always labeled A, B, C, and so on to prevent bias in result interpretation and CFU acquisition. When appropriate, CFU were counted by an individual that had no information on the treatment groups.
4.b. For animal studies, include a statement about blinding even if no blinding was done	All mice experiments involving Citrobacter rodentium were performed using a blind study design.
5. For every figure, are statistical tests justified as appropriate?	Yes, One-way ANOVA was performed to verify statistical significance in CRISPR killing experiment performed under laboratory conditions (Figure EV1, EV4). Alpha and beta variance analysis were also performed to evaluate the impact of the COP treatment on the mice microbiome in 16S ribotyping experiment, which are widely used markers of dysbiosis (Appendix Figure S2). PERMANOVA was used to calculate the corrected statistical significance for any difference found in beta diversity (Appendix Figure S2h).

Do the data meet the assumptions of the tests (e.g., normal distribution)? Describe any methods used to assess it.	For killing experiments as well as conjugation efficiency data, raw data are typically distributed in a log normal manner. To allow the use of One-way ANOVA on these data, a log transformation was used prior to the test. For statistical tests performed on the metagenomics data, the Qiime2 software, which is widely used for metagenomics analysis, was used for all calculation. Beta diversity distribution were fit for the PERMANOVA q-value test.
Is there an estimate of variation within each group of data?	Yes, SD (Figure 2c, 4a, 4b-f lower panels, EV1, EV3, S1a), median (Figure 1, 3, 4, EV2, S1a, S2), interquartile range (Figure 1, 3, 4, EV2, S2) and individual data points (Figure 1, 2, 3, 4, EV1, EV2, EV4, S1ab) are shown.
Is the variance similar between the groups that are being statistically compared?	Yes

C- Reagents

6. To show that antibodies were profiled for use in the system under study (assay and species), provide a citation, catalog number and/or clone number, supplementary information or reference to an antibody validation profile. e.g., Antibodypedia (see link list at top right), 1DegreeBio (see link list at top right).	NA
7. Identify the source of cell lines and report if they were recently authenticated (e.g., by STR profiling) and tested for mycoplasma contamination.	NA

* for all hyperlinks, please see the table at the top right of the document

D- Animal Models

8. Report species, strain, gender, age of animals and genetic modification status where applicable. Please detail housing and husbandry conditions and the source of animals.	Mice, C57 BL6, Female, of 16-18 grams were used.
9. For experiments involving live vertebrates, include a statement of compliance with ethical regulations and identify the committee(s) approving the experiments.	All mice-related protocols were strictly evaluated to avoid animal suffering by the Université de Sherbrooke Animal Care Committee.
10. We recommend consulting the ARRIVE guidelines (see link list at top right) (PLoS Biol. 8(6), e1000412, 2010) to ensure that other relevant aspects of animal studies are adequately reported. See author guidelines, under 'Reporting Guidelines'. See also: NIH (see link list at top right) and MRC (see link list at top right) recommendations. Please confirm compliance.	All relevant informations are stated in the manuscript.

E- Human Subjects

11. Identify the committee(s) approving the study protocol.	NA
12. Include a statement confirming that informed consent was obtained from all subjects and that the experiments conformed to the principles set out in the WMA Declaration of Helsinki and the Department of Health and Human Services Belmont Report.	NA
13. For publication of patient photos, include a statement confirming that consent to publish was obtained.	NA
14. Report any restrictions on the availability (and/or on the use) of human data or samples.	NA
15. Report the clinical trial registration number (at ClinicalTrials.gov or equivalent), where applicable.	NA
16. For phase II and III randomized controlled trials, please refer to the CONSORT flow diagram (see link list at top right) and submit the CONSORT checklist (see link list at top right) with your submission. See author guidelines, under 'Reporting Guidelines'. Please confirm you have submitted this list.	NA
17. For tumor marker prognostic studies, we recommend that you follow the REMARK reporting guidelines (see link list at top right). See author guidelines, under 'Reporting Guidelines'. Please confirm you have followed these guidelines.	NA

F- Data Accessibility

18: Provide a "Data Availability" section at the end of the Materials & Methods, listing the accession codes for data generated in this study and deposited in a public database (e.g. RNA-Seq data: Gene Expression Omnibus GSE39462, Proteomics data: PRIDE PXD000208 etc.) Please refer to our author guidelines for 'Data Deposition'.<br><br>Data deposition in a public repository is mandatory for:<br>a. Protein, DNA and RNA sequences<br>b. Macromolecular structures<br>c. Crystallographic data for small molecules<br>d. Functional genomics data<br>e. Proteomics and molecular interactions	A Data Availability section is present in the manuscript and lists all accession numbers for datasets supporting the figures: Data availability:<br>Reads for COP escapers analysis, 16S ribotyping, and mutagenesis experiments are available at NCBI under BioProject: PRJNA510637, PRJNA510842, and PRJNA674373 respectively. Raw CFU counts and conjugation frequency evaluation are available upon request.
19. Deposition is strongly recommended for any datasets that are central and integral to the study; please consider the journal's data policy. If no structured public repository exists for a given data type, we encourage the provision of datasets in the manuscript as a Supplementary Document (see author guidelines under 'Expanded View' or in unstructured repositories such as Dryad (see link list at top right) or Figshare (see link list at top right).	All relevant data were submitted to public repository.
20. Access to human clinical and genomic datasets should be provided with as few restrictions as possible while respecting ethical obligations to the patients and relevant medical and legal issues. If practically possible and compatible with the individual consent agreement used in the study, such data should be deposited in one of the major public access-controlled repositories such as dbGAP (see link list at top right) or EGA (see link list at top right).	NA
21. Computational models that are central and integral to a study should be shared without restrictions and provided in a machine-readable form. The relevant accession numbers or links should be provided. When possible, standardized format (SBML, CellML) should be used instead of scripts (e.g. MATLAB). Authors are strongly encouraged to follow the MIRIAM guidelines (see link list at top right) and deposit their model in a public database such as Biomodels (see link list at top right) or JWS Online (see link list at top right). If computer source code is provided with the paper, it should be deposited in a public repository or included in supplementary information.	NA

G- Dual use research of concern

22. Could your study fall under dual use research restrictions? Please check biosecurity documents (see link list at top right) and list of select agents and toxins (APHIS/CDC) (see link list at top right). According to our biosecurity guidelines, provide a statement only if it could.	We do not anticipate the data presented in the present manuscript to fall under dual use research retrictions.
